# Use of Deep Sequencing to Evaluate Transitions in Microbial Communities in Stranded *Sargassum*

**DOI:** 10.1155/ijm/3915271

**Published:** 2025-07-21

**Authors:** Afeefa A. Abdool-Ghany, Kristina M. Babler, David Bogumil, Sarah Pollock, Jiayu Li, Schonna R. Manning, Helena M. Solo-Gabriele

**Affiliations:** ^1^Department of Chemical, Environmental, and Materials Engineering, University of Miami, Coral Gables, Florida, USA; ^2^Department of Planning and Analytics, Brizaga Inc., Fort Lauderdale, Florida, USA; ^3^Department of Biological Sciences, Institute of Environment, Florida International University, North Miami, Florida, USA; ^4^Utah Public Health Laboratory, Taylorsville, Utah, USA; ^5^Department of Sequencing Operations, Ultima Genomics Inc., Fremont, California, USA; ^6^Department of Mechanical and Aerospace Engineering, University of Miami, Coral Gables, Florida, USA

**Keywords:** decomposition, deep sequencing, fecal coliform bacteria, microbial communities, *Sargassum*, stranding, *Vibrio*

## Abstract

Deep sequencing technologies can be used to evaluate pathogens in environmental samples. The objective of this study was to use this technology to evaluate *Sargassum* samples that were characterized by different stranding times, one classified as short-term stranded (STS) and another classified as long-term stranded (LTS) *Sargassum*. Nine replicates of the STS *Sargassum* showed a range in Shannon diversity between 3.04 and 3.38, whereas 11 replicates of LTS showed a range between 1.17 and 1.22. Nonmetric multidimensional scaling analysis showed distinct differences between STS and LTS by about 0.5 coordinate units, while variations within replicates ranged by 0.1 coordinate units. Comparison between the two *Sargassum* samples showed a greater abundance of *Vibrio* species in STS *Sargassum* when compared to LTS *Sargassum*, with major pathogenic forms observed for *Vibrio alginolyticus* (11%), *Vibrio parahaemolyticus* (1.5%), and *Vibrio vulnificus* (0.29%). Additional known human pathogens were observed, including *Listeria monocytogenes*, *Legionella pneumophila*, and *Staphylococcus aureus*, as well as the presence of gut commensals and fecal coliforms. Overall results show that deep sequencing analysis of these environmental samples was reproducible. Given the abundance of pathogenic bacteria, more research is needed to evaluate the risk of disease transmission as *Sargassum* strands and decomposes on coastal beaches.

## 1. Introduction

In addition to lower costs per sequenced base [1], sequencing technologies have evolved in their sensitivities, allowing for taxonomic analysis of heterogeneous environmental samples down to the species level. These improvements have made it possible to characterize microbial communities in complex environmental samples at an affordable cost. Environmental samples are typically analyzed at different points in time and space. When sequencing these samples, the sequencing is typically conducted once. Rarely is sequencing replicated, which is necessary to understand the reproducibility of the technology. In this study, we set out to replicate two environmental samples^∗^.

1

The two environmental samples chosen for this study consisted of *Sargassum*, a genetically diverse genus [2] of brown macroalgae. We chose to evaluate *Sargassum* due to its recent increase in coastal influxes, calling into question potential human health impacts. The most common forms of *Sargassum* in South Florida include *Sargassum fluitans III* and *Sargassum natans I* [3]. Since 2011, *Sargassum* has been known to inundate the shores of the Caribbean, western Africa, and coastal cities of the United States [4–6], with quantities showing an increase in the last decade, presumably due to changes in climate and nutrient deliveries to the Atlantic Ocean [7–9]. Furthermore, there has been a continual rise in biomass each year, as shown by research tracking the onshore influx of *Sargassum* [10].

One area of concern associated with the influx is the potential for *Sargassum* to harbor human pathogens. From a microbiological perspective, holopelagic *Sargassum* has been shown to serve as a crucial microbial substrate, releasing organic matter and promoting microbial colonization [11]. Research on its microbiome is limited, but studies indicate a diverse community, including photosynthetic and nitrogen-fixing bacteria such as *Rhodobacteraceae* and *Cyanobacteria* [12]. The microbiome varies among distinct *Sargassum* genotypes, and elevated relative abundances of *Vibrio* and *Alteromonas* have been linked, including certain *Vibrio* operational taxonomic units (OTUs) identified as potential pathogens [13, 14]. As *Sargassum* is stranded and decomposes on the coast, it can introduce associated bacteria and serve as a substrate for pathogens, impacting coastal ecosystems [15]. Previous studies have shown an increase in culturable fecal indicator bacteria, enterococci, and fecal coliform, as seaweed decomposes on the beach [16, 17].

The objective of this study was to use deep-sequencing technologies to evaluate *Sargassum* samples that were characterized by different stranding times. Although microbial communities have been measured at the genus level for oceanic free-floating *Sargassum*, limited studies have been conducted on stranded *Sargassum* that documented high diversity at the family level [13]. Even fewer studies have been conducted on the microbial communities at the species level and the presence of fecal indicator bacteria. A comparison was made between short-term stranded (STS) *Sargassum* versus long-term stranded (LTS) *Sargassum*. This study is unique in the number of replicates of deep sequencing analyses on the same environmental samples, in the depth of sequencing corresponding to an average of 125 million reads per analysis, and in the advanced bioinformatics used to provide information at the species level. This is the first study to our knowledge to evaluate species-level microbial communities in stranded *Sargassum*. The results of this study are significant in understanding the reproducibility of deep-sequencing technology. Results can also be used as a starting point to assess the potential human health impacts of stranded *Sargassum*.

## 2. Materials and Methods

### 2.1. *Sargassum* Collection

**Figure 1:**
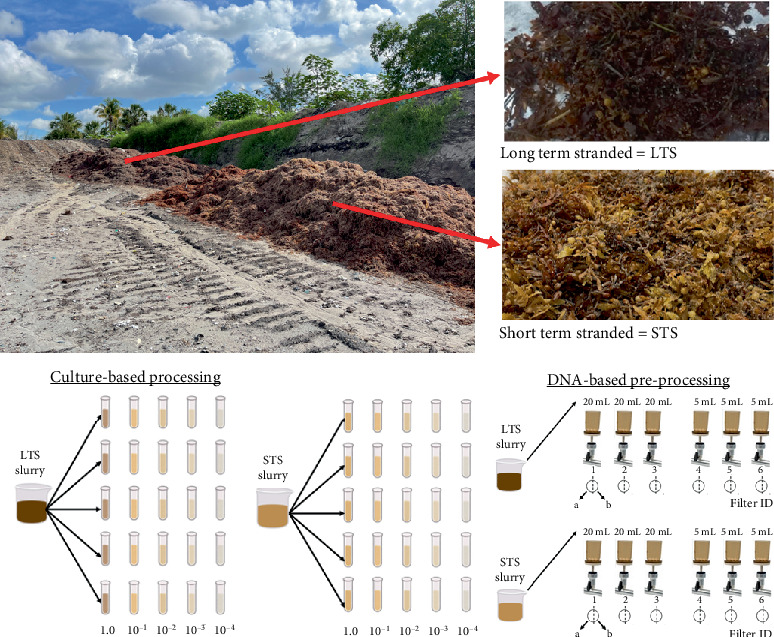
Example of STS and LTS Sargassum samples found at the staging area of the community compost pile (top photos) and illustration of the experimental design of the culture-based fecal coliform processing (bottom left inset) and for the DNA preprocessing of samples (bottom right inset).

Two samples of *Sargassum*, which consisted mostly of *Sargassum fluitans III* and *Sargassum natans I*, were collected from a *Sargassum* composting facility in Broward County, Florida, United States (26°4⁣′57.7704⁣^″^ N, 80°8⁣′53.466⁣^″^ W). In this study, we did not separate the *Sargassum* samples between these two different species as we did not expect interspecies differences in microbial communities. These samples were originally collected from the intertidal zone using a beach raker and hauled to the compost site located within 5 km of the beach. Samples from the compost pile were collected aseptically into sterile Whirl-Pak bags and transported in coolers with ice packs. They were classified based on their time spent in the composting pile. This study was limited to two samples as the focus was on assessing the reproducibility of the sequencing results. The selected samples allowed us to evaluate the consistency of microbial community profiles under controlled conditions, minimizing variability from external environmental factors. The *Sargassum* retrieved same-day from the beach by the beach groomer was considered STS, having been stranded for less than 24 h before collection (Figure 1). The LTS samples, on the other hand, had been at the composting facility for 3 days before collection, undergoing decomposition (Figure 1). Collection took place on June 20, 2022, with an average air temperature of 28°C and no precipitation observed. In the week leading up to the collection, the average air temperature was 29°C. Three days prior to sample collection, the cumulative rainfall was 3.23 cm.

### 2.2. *Sargassum* Preprocessing

Upon receipt at the laboratory, *Sargassum* samples were split. The first split was processed immediately for moisture content and, to confirm culturable fecal bacteria, this first split was also analyzed for fecal coliform. The second split was frozen for subsequent DNA sequencing analysis. Preprocessing for both portions used the procedure described by Abdool-Ghany et al. [16]. In brief, the *Sargassum* sample that was immediately processed was first homogenized by placing it on a sterile tray and using sterile scissors to cut the *Sargassum* into about 2-cm pieces and mixing during the cutting process. One aliquot of the cut and homogenized *Sargassum* was analyzed gravimetrically for moisture content (110°C drying temperature for 24 h) to report results on a *Sargassum* dry-weight basis. Then, 10 g of the cut seaweed was aseptically transferred to a sterile Whirl-Pak bag containing 200 mL of sterile phosphate-buffered saline (PBS). The seaweed was mixed by rubbing the bag between the thumb and fingertips for 2 min to release the microbes from the *Sargassum* surface. This slurry mixture was allowed to settle for 2 min before aliquots were drawn for fecal coliform analysis as described below. For DNA sequencing, the *Sargassum* was thawed on ice packs and then preprocessed to generate the slurry mixture in the same manner as for the sample processed immediately^∗^.

2

### 2.3. Culture-Based Fecal Coliform Analysis

The presence and abundance of fecal coliform bacteria were analyzed for the *Sargassum* slurry mixture by multiple-tube fermentation as per standard methods used to assess Class A biosolids (Method 1681, US EPA 2006 [18]). In brief, the procedure involved preparing dilutions (e.g., 1.0, 10^−1^, 10^−2^, 10^−3^, and 10^−4^) of the slurry mixture using sterile PBS. Five series of five tubes each containing 10 mL of media were prepared. The first series of five tubes contained double-strength (2X) A1-media to which 10 mL of a 10^−1^ dilution was added. Series two to five contained single-strength (1X) A1-media and received 1 mL from the 10^−1^, 10^−2^, 10^−3^, and 10^−4^ dilutions. Tubes were incubated at 35°C for 3 h and then transferred to a water bath at 44.5°C and incubated for an additional 21 h.

### 2.4. DNA Preprocessing

Samples for DNA sequencing were prepared by filtering slurry mixtures through GN-6 Metricel filter membranes (47 mm, 0.45 *μ*m, Pall Corp. 148 #66278). Six filters were used for STS and for LTS *Sargassum* samples, resulting in a total of 12 filters to assess the reproducibility of sequencing. Sample volumes for filtration were 20 mL for the first three filters (1, 2, and 3) of each sample type and 5 mL for the last three filters (4, 5, and 6). Filters were then placed on sterile dishes, cut in half (half “a” and half “b”) using a sterile scalpel due to space limitations of the tubes available for the next step. Each half filter was folded and inserted into separate sterile Zymo ZR BashingBead Lysis tubes (containing 0.1- and 0.5-mm beads, Cat#S6012-50) with 1 mL of DNA/RNA shield [19–21]. These tubes, a total of 12 for each sample type, were kept at 4°C until DNA extraction. The “a” and “b” paired tubes were true replicates. The 1, 2, and 3 were replicate filters of heavily concentrated particulates (20 mL) whereas 4, 5, and 6 represented filter replicates with less concentrated particulates (5 mL).

### 2.5. DNA Extraction

The commercial kit used for DNA extraction (ZymoBIOMICS DNA Miniprep kit, Zymo Research Cat. # D4300) was optimized in prior studies for wastewater analyses [19]. In brief, the 24 lysis tubes were placed on a bead beater (OMNI Bead-Ruptor12), with 400 *μ*L of the bashed seaweed concentrate transferred directly into the first column of the ZymoBIOMICS Miniprep kit and processed according to the manufacturer's recommendations for DNA extraction, except for the increase in the initial centrifugation step from 8000 × *g* to 10,000 × *g* to improve the pelleting of ruptured membrane particles [19]. Nucleic acid concentrations (nanograms per microliter) were assessed in duplicate on a Synergy BioTek Plate Reader using 2 *μ*L of purified nucleic acid per sample well; two nuclease-free water blanks were used per 16-well plate as negative controls.

### 2.6. Sequencing

PCR-free libraries were generated starting with approximately 500 ng of genomic DNA for each of the 24 extracts. The DNA was enzymatically fragmented, end-repaired, and poly A-tailed using the NEBNext Ultra II FS DNA Library Prep Kit (New England Biolabs). xGEN PCR-free adapters for Ultima Genomics (Integrated DNA Technologies) were ligated, and the resulting library molecules were cleaned up using a double-sided SPRI magnetic bead technology with average insert sizes of approximately 350–400 bp. Library size was determined by running 1 *μ*L on a High Sensitivity D1000 TapeStation (Agilent Technologies), and the library was quantified using the NEBNext Library Quantification Kit (New England Biolabs). Ultima Genomics processed the samples for sequencing analysis. Whole-genome shotgun sequencing was performed on the Ultima Genomics (UG) V100 platform using an open-flow cell design on a circular wafer with a large surface area and mostly natural nucleotides that allow optical end-point detection without reversible terminators [22]. Sample pools were then seeded onto UG sequencing beads, pre-enriched, and amplified by emulsion PCR, allowing for automated sequencing bead preparation. Sequencing was performed on UG100 sequencing systems, running 464 flow cycles, 116 cycles across each of the four nucleotides (A, G, T, and C).

### 2.7. Bioinformatics Analyses

Reads were aligned to the human genome assembly GRCh38/hg38 using BWA MEM [23]. Reads not aligning to the human genome were extracted from the BAM alignment and converted to fastq using samtools v1.11 [24]. All nonamplifying UG primers, adapters, and barcodes were trimmed on the UG100 sequencer. Taxonomic annotation of these reads was performed using kraken2 v2.1.2 [25] with the PlusPFP database (standard plus protozoa, fungi, and plant version from 12/09/2022; https://genome-idx.s3.amazonaws.com/kraken/k2_pluspfp_20221209.tar.gz). To minimize false positives while maintaining sensitivity, we applied a confidence score threshold of ≥ 0.1. Read quality was assessed using the fraction of bases with quality ≥ 20 and the mean read length for each library. In addition, the UG100 runs a human DNA spike control in each run; statistics were within normal parameters. Contamination of human DNA was removed by discarding all reads mapped to the human genome GRCh38/hg38. Primary results from the bioinformatics analysis include relative abundance among the microbes observed in the samples. Given the depth of the analyses, abundances are reported in parts per million instead of the more common unit of percent.

### 2.8. Statistical Analysis

Reproducibility was assessed by comparing the range of Shannon indices among replicate extracts and by examining the range of clustering in nonmetric multidimensional analysis (nMDS) analysis. Shannon indices were calculated for each replicate extract using the statistical software PAST (Version 4.01). nMDS was conducted on the sequencing data to assess the similarities between the sample types. Bray–Curtis was used as the similarity index. To test the significance of differences between the sample types, an analysis of similarities (ANOSIMs) test was conducted. Similarity percentages (SIMPERs) were also used to determine the species that contributed most to the average dissimilarity of the sample types. The range of clustering within replicates and between samples was assessed by evaluating the differences in the nMDS plot coordinates.

When interpreting the results from the sequencing analysis, species-level data was categorized into genus-level categories of known gut commensals (*Bacteroides*, *Bifidobacterium*, *Clostridium*, *Eubacterium*, *Enterococcus*, and *Lactobacillus*; [26]) (see Table 1 for list), fecal coliform bacteria (*Citrobacter*, *Enterobacter*, *Escherichia*, and *Klebsiella*; *ibid*), and additional genera known to contain pathogenic forms (see Table 2 for list). Abundance data were analyzed in R v4.3.2 with ComplexHeatmap v2.15.4.

## 3. Results

### 3.1. Culture-Based Fecal Coliform Analysis

Higher concentrations of fecal coliforms were observed in the STS *Sargassum* samples (41 most probable number [MPN] per dry gram) when compared to the LTS samples (< 18 MPN per dry gram). Moisture contents for the STS and LTS samples were 51% and 9%, respectively.

### 3.2. Sequencing

In total, of the 24 DNA extracts, only 20 were fully sequenced. Four extracts were excluded from the analysis due to failed sequencing; these included three STS samples and one LTS sample. The total number of reads for the STS extracts was 37,814,199, whereas the total number of reads for the LTS extracts was 166,126,896, representing nearly 4.5× more reads. The majority (over 90%) of the reads were found to be within the domain bacteria, so the analysis focused on bacterial species. Raw sequencing data, following the removal of human reads and trimming, are available through the Sequence Read Archive (SRA) database using the following accession number: PRJNA1093391.

**Figure 2:**
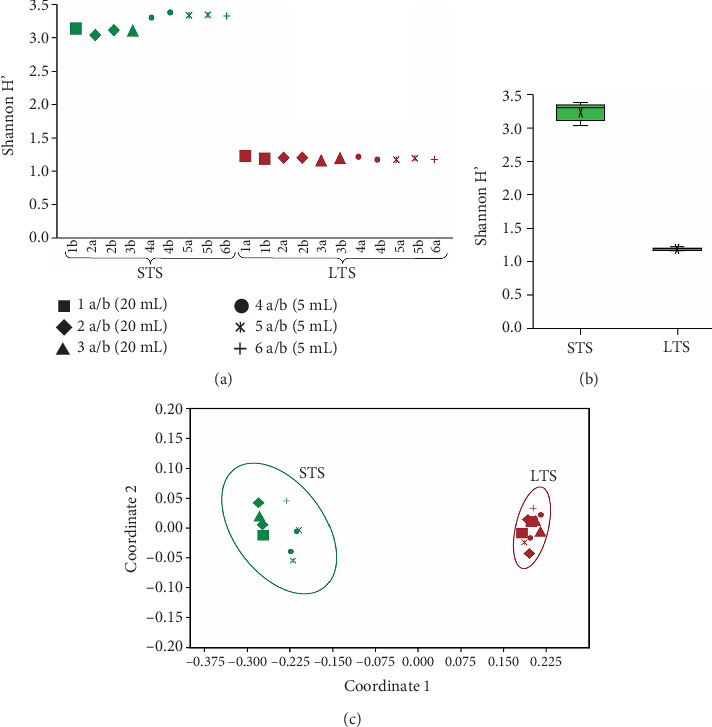
(a) Shannon diversity index of individual extract of the STS and LTS samples at the species level. The larger symbols represent a larger volume that was passed through the filter, while the smaller symbols represent a smaller volume passed through the filter. (b) Shannon diversity index of the STS and LTS samples at the species level. (c) nMDS plot of replicate samples across STS and LTS samples.

Among the 20 extracts, 6065 unique bacterial species were identified, with 5291 bacterial species identified for STS *Sargassum* and 5365 bacterial species identified for LTS *Sargassum*. Diversity was significantly different across sample types (Kruskal–Wallis test, *p* < 0.001 for species-level comparisons). Diversity was higher in STS *Sargassum*, with a mean Shannon diversity index (SI) of 3.23 when compared to the decomposing LTS *Sargassum* that had an SI of 1.19 (Figure 2a,b). Within sample types, diversity ranged more for the STS sample (SI from 3.039 to 3.377, CV = 3.8%) than for the LTS sample (SI from 1.165 to 1.224, CV = 1.6%). Of interest was the shift in diversity for the 4, 5, and 6 filters (5 mL filtration volume) in comparison to the 1, 2, and 3 filters (20 mL filtration volume), especially in the STS samples (*p* < 0.001). This shift was not observed for the LTS samples, indicating that filtration volumes did not impact SI for this sample (*p* = 0.40).

**Figure 3:**
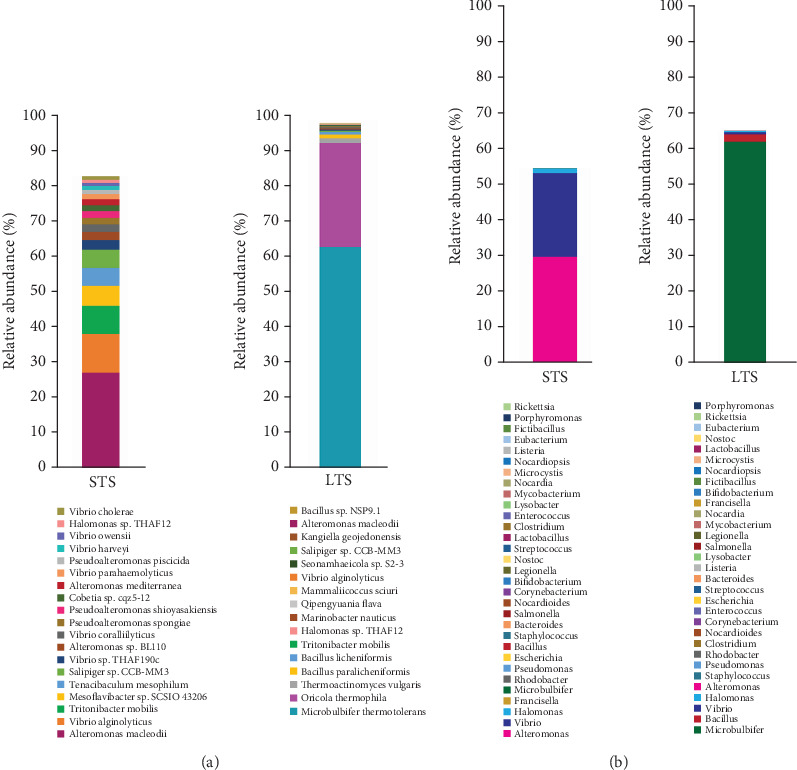
Comparison of (a) species level and (b) genus level results. Most abundant species in STS and LTS samples are shown in (a), and most abundant genera in STS and LTS samples are shown in (b).

nMDS analysis of the successful 20 sequencing runs showed clear distinctions among sample types (Figure 2c). The nMDS ordination is supported by the ANOSIM test that also showed significant differences across sample types (*p* < 0.001). SIMPER analysis showed four bacterial species contributed the most to dissimilarity between the STS and LTS samples: *Microbulbifer thermotolerans* (50.9%), *Oricola thermophila* (24.0%), *Alteromonas macleodii* (6.1%), and *Vibrio alginolyticus* (2.4%). The thermotolerant species of *M. thermotolerans* and *O. thermophila* were abundant in the LTS *Sargassum*, whereas *A. macleodii* and *V. alginolyticus* were abundant in the STS *Sargassum*. Additional bacterial species abundant in STS and LTS *Sargassum* are shown in Figure 3a with additional details in Tables S1–S27 in the supporting information.

Within a sample type, there was some spread among the coordinates within the nMDS plot. The coordinates of the STS sample varied more than the coordinates of the LTS sample, which clustered more closely. Between each sample type, the average of Coordinate 1 was −0.244 ± −0.029 units, whereas the average for Coordinate 2 was −0.0010 ± −0.032. Within the STS replicates, the average of Coordinate 1 was −0.218 ± 0.006 units, with data corresponding to Filters 4, 5, and 6 (5 mL filtration volume) shifted horizontally with an average of −0.277 ± 0.021 units relative to data corresponding to Filters 1, 2, and 3 (20 mL). For LTS, the coordinates overlapped more closely, with Coordinate 1 differing by only 0.004 units, with no effect of filtration volume observed. Overall, the variation among the STS samples was small in relation to the differences between STS and LTS samples (Figure 2c).

**Figure 4:**
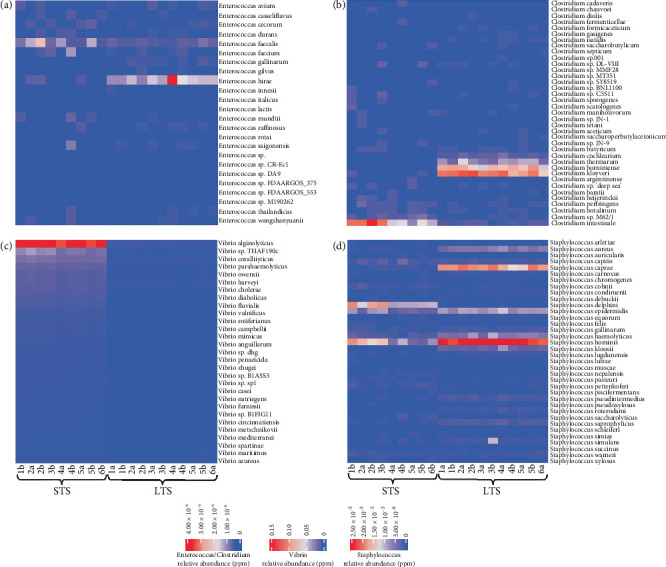
Heat maps of species abundances across the STS and LTS *Sargassum* samples. (a) Species of *Enterococcus* (24) that were identified. (b) Species of *Clostridium* (37) that were identified. (c) Top 29 of 80 species of *Vibrio*; only *Vibrio* species that exhibited an abundance of greater than 0.2 ppm were plotted. (d) Species of *Staphylococcus* (36) that were identified.

To illustrate the potential value of deep sequencing results, we observe that among the gut commensals and fecal coliform bacteria, as shown in Table 1, the number of species identified within each genus varied. Notable among this group was the identification of species-level fecal indicator bacteria that make up the enterococci group. *Enterococcus faecalis* was found at an abundance of 0.95 ppm in the STS *Sargassum* and *E. hirae* at an abundance of 1.61 ppm in the LTS *Sargassum* (Figure 4a). For *Clostridium*, among the 35 species that were identified, the most abundant species in the STS sample was *C. intestinale* (1.98 ppm), while in the LTS samples, *C. kluyveri* was the most abundant (2.51 ppm) (Figure 4b). *Bacteroides uniformis*, *Bifidobacterium adolescentis*, and *Lactobacillus crispatus* dominated both the STS and LTS *Sargassum* within their corresponding genus, respectively.

Among the fecal coliforms, *Klebsiella pneumoniae* was the most abundant species within the *Klebsiella* genus in both the STS *Sargassum* (140 ppm) and the LTS *Sargassum* (11.8 ppm). Similarly, among the five species of *Escherichia*, *Escherichia coli* was the most abundant (STS *Sargassum* = 40.2 ppm, LTS *Sargassum* = 1.49 ppm). Different species were most abundant among *Citrobacter* and *Enterobacter.* Overall, abundances in STS were higher than in LTS.

When analyzing the genus-level data of pathogen-specific bacteria, there were 31 genera containing potentially pathogenic species (Figure 3). Overall, it was noted there was a higher abundance of *Vibrio* species within the STS samples as opposed to the LTS samples. Among the 80 species of *Vibrio* identified (Figure 4 and Table 2), the most abundant species was *V. alginolyticus*, which exhibited the highest abundance in the STS samples, averaging 110,000 ppm (representing 11% among all reads inclusive of all genera). In contrast, the abundance of *V. alginolyticus* in the LTS samples was 2000 ppm (0.2%), on average. Moreover, four additional pathogenic *Vibrio* species were identified in both the STS and LTS samples (Table 2). Noteworthy among these, *Vibrio vulnificus* (2900 and 26.3 ppm), *Vibrio cholerae* (9300 and 190 ppm), and *Vibrio parahaemolyticus* (15,000 and 180 ppm) were found in STS and LTS *Sargassum*, respectively.

Additional pathogenic bacteria of interest that were generally higher in the STS *Sargassum* included *Shigella flexneri* (0.49 ppm), *Salmonella enterica* (5.69 ppm), *Legionella pneumophila* (1.06 ppm), *Corynebacterium diphtheriae* (0.12 ppm), and *Francisella tularensis* (104 ppm). By comparison, notable pathogens that were generally higher in LTS *Sargassum* included *Staphylococcus aureus* (5.54 ppm) (Figure 4d) and *Listeria monocytogenes* (1.21 ppm).

## 4. Discussion

In this study, the microbial communities of STS and LTS *Sargassum* were compared for species-level microbial community composition using next-generation sequencing (NGS) with deep-sequencing methods. STS and LTS samples were chosen to represent the range of *Sargassum* to which beach-goers would be potentially exposed, with STS representing freshly beached material and LTS representing *Sargassum* that has remained onshore for an extended period. Including LTS samples provides insight into how microbial communities, particularly those of public health concern, may shift over time as *Sargassum* degrades and is exposed to environmental conditions such as UV radiation, desiccation, and temperature fluctuations. These environmental stressors could contribute to a decline in viable fecal indicator bacteria and associated DNA integrity. This distinction is important for interpreting public health risk, as degraded DNA may still be detected by sequencing but may not represent viable or infectious organisms. The nine STS replicates and the 11 LTS replicates illustrate the degree to which sequencing results are expected to be reproducible. Although distinct differences were observed in SI and mMDS analysis among the two samples, results showed that filtration volume resulted in a shift (although small) in sequencing results. The shift was observed for one of the samples (STS) but not for the other (LTS), suggesting that optimization of sample preprocessing is sample-dependent. Our true replicates, the “a” and “b” filters, and our filter replicates showed good reproducibility consistent with studies that have shown that reproducibility across sample replicates and sequencing flow cells is acceptable [27, 28]. Given the differences observed among the STS samples among different filtration volumes, studies should include experimentation that evaluates the changes of sample preprocessing on downstream sequencing results.

The results of this study also demonstrated the utility of NGS for microbiome characterization. Unlike 16S rRNA sequencing, which primarily targets a specific bacterial genome region, NGS provides a more comprehensive perspective by sequencing the entire genetic material of a sample [29]. This broader approach not only captures bacterial diversity but also encompasses archaea, viruses, fungi, and microeukaryotes [30]. Moreover, NGS can detect functional genes and metabolic pathways, enabling insights into the functional potential and ecological roles of the microbial community [1, 31, 32]. Although this study focused on bacterial results, NGS revealed evidence of fungi and, to a lesser extent, archaea within the reads, despite over 90% of the reads corresponding to bacteria. To obtain a more holistic understanding of microbial communities in stranded *Sargassum*, future efforts should emphasize evaluating viruses and microbes from other domains of life.

Among the decomposing *Sargassum* analyzed in the current study, the highest levels of cultured fecal coliform bacteria were observed among the STS *Sargassum*, with lower levels within the LTS. This difference in cultured fecal coliform was consistent with the lower percent abundances for most (but not all) pathogenic species observed in the stranded *Sargassum*. The lower abundance of fecal bacteria observed in LTS may be due to DNA degradation as the *Sargassum* dries.

Overall data showed that stranded *Sargassum* potentially harbors gut commensals, fecal coliforms, and known pathogenic bacteria. The presence of so many different gut commensals and fecal coliform species suggests the introduction of fecal sources. Some of the species identified have been associated with gastrointestinal ailments, including *E. coli*, *E. faecalis*, and *K. pneumoniae*. Fecal-associated bacteria can come from the coast and shoreline [33], through bird feces [34], dog feces [33], and human bather shedding [35–37]. Results suggest that as the stranded *Sargassum* decomposes, its microbial community changes, generally containing lower abundances of the fecal bacteria highlighted in the results. However, the LTS *Sargassum* showed measurable levels of all listed microbes, with some notable microbes at higher levels (e.g., *Clostridium* sp.) than observed in STS *Sargassum*.

Among the pathogenic species evaluated, *Vibrio* is the most notorious in the context of *Sargassum*. Species of *Vibrio* are recognized for their nonpathogenic forms and their pathogenic forms. They are adaptable and hold many roles within the marine ecosystem. Various species of *Vibrio* aid in the role of light-emitting symbioses, facultative fermentation, and nutrient acquisition [11, 38]. Sargassum-associated plankton could also potentially affect the abundance and distribution of *Vibrio* species, including those with clinical significance [39].

Among the pathogenic forms, common species that cause human illness include *V. parahaemolyticus* (STS, 15,000 ppm; LTS, 180 ppm), *V. vulnificus* (STS, 2900 ppm; LTS, 26.3 ppm), and *V. alginolyticus* (STS, 110,000 ppm; STS, 2000 ppm). These three species are in the Top 10 species of *Vibrio* in the STS samples. Among the three, *V. vulnificus* is particularly of concern for its ability to cause wound infections among bathers [40]. *V. vulnificus* infections are tracked in Florida, showing an increase in fatalities over time, especially during extreme flooding events such as those that occur immediately after hurricanes [41]. Compared to the LTS samples, the *Vibrio* species in STS samples were less abundant, suggesting that drying of *Sargassum* may assist in reducing disease risks. Salinity is also known to impact the prevalence of *V. vulnificus* [42], a factor that can also be used to control risks. Since the detection of nucleic acids of pathogens does not necessarily translate to viable microbes capable of infection, studies are therefore needed to determine whether the detection of pathogenic *Vibrio* species found in stranded *Sargassum* is capable of infecting humans.

In contrast to *Vibrio*, which declined in the LTS *Sargassum*, *S. aureus* was observed in higher abundances in LTS compared to STS *Sargassum*. The presence of *S. aureus* has been documented by others in beach environments and has been attributed to human bather shedding [43, 44]. These results suggest that *S. aureus* may also be associated with decomposing *Sargassum* in addition to direct human sources.

Overall, the detection of pathogenic species of bacteria within LTS or STS *Sargassum* is notable and warrants additional study to confirm the presence of these pathogenic species through targeted PCR analysis and through culture-based analysis methods to determine the potential viability of these pathogens. Measurement of virulence factors known to be involved in infections may also provide information about potential health implications [45]. Risk assessments should be conducted with this additional information to determine whether populations that come into contact with stranded *Sargassum* are at risk.

The results from this study are also of value from a microbial ecology perspective, which has implications for human health, as *Sargassum* is known to accumulate metals, in particular arsenic, and other pollutants [46–49]. For example, we observed bacteria that impart arsenic resistance (*Fictibacillus arsenicus*, [50]). In addition, as *Sargassum* decomposes, it produces high levels of enterococci [16] and produces hydrogen sulfide and ammonia [51]. Much more work is needed to understand these threats mediated by microbes, inclusive of hydrogen sulfide generation, release of arsenic during degradation, and the impacts of the microbial community on pathogen levels and risks to human health. NGS results provided in this study emphasize the reproducibility of sequencing data and provide some of the first detailed insights into the changing microbial community of stranded *Sargassum* that can have potential consequences on human health.

## Figures and Tables

**Table 1:** Most abundant forms of gut commensals (*Bacteroides*, *Bifidobacterium*, *Clostridium*, *Eubacterium*, *Enterococcus*, and *Lactobacillus*) and fecal coliforms (*Citrobacter*, *Enterobacter*, *Escherichia*, and *Klebsiella*) from the STS and LTS samples of *Sargassum*.

		**STS**		**LTS**	
**Genus**	**Number of ** **species**	**Most abundant ** **and/or pathogenic ** **forms**	**Abundance ** **(ppm)**	**Most abundant ** **and/or pathogenic ** **forms**	**Abundance ** **(ppm)**
Gut commensals					
*Bacteroides*	25	*B. uniformis*	4.65	*B. uniformis*	0.32
*Bifidobacterium*	18	*B. adolescentis*	2.81	*B. adolescentis*	0.11
*Clostridium*	35	*C. intestinale*	1.98	*C. kluyveri*	2.51
*Eubacterium*	4	*E. sp. NSJ-61*	0.05	*E. sp. MSJ-33*	0.02
*Enterococcus*	15	*E. faecalis*	0.95	*E. hirae*	1.61
*Lactobacillus*	18	*L. crispatus*	2.08	*L. crispatus*	0.11
Fecal coliform					
*Citrobacter*	25	*C. freundii*	9.11	*C. braakii*	0.29
*Enterobacter*	34	*E. hormaechei*	6.42	*E. kobei*	1.45
*Escherichia*	5	*E. coli*	40.2	*E. coli*	1.49
*Klebsiella*	16	*K. pneumoniae*	140	*K. pneumoniae*	11.8

**Table 2:** Most abundant bacterial pathogens present in fresh (STS) and decomposing (LTS) samples of *Sargassum*.

**Pathogens on *Sargassum***					
		**STS**		**LTS**	
**Genus**	**Number of Species**	**Most abundant and/or pathogenic forms**	**Value of abundance (ppm)**	**Most abundant and/or pathogenic forms**	**Value of abundance (ppm)**
*Campylobacter*	25	*C. concisus*	1.37	*C. concisus*	0.05
*Corynebacterium*	67	*C. diphtheriae* *C. tuberculostearicum*	0.12 5.37	*C. diphtheriae* *C. stationis*	0.03 0.88
*Fictibacillus*	2	*F. arsenicus* *F. phosphorivorans*	0.02 0.34	*F. arsenicus* *F. phosphorivorans*	0.13 0.25
*Francisella*	15	*F. tularensis*	104	*F. tularensis*	0.75
*Legionella*	19	*L. pneumophila* *L. sainthelensi*	1.06 2.10	*L. pneumophila* *L. israelensis*	0.03 0.43
*Listeria*	8	*L. monocytogenes*	0.24	*L. monocytogenes*	1.21
*Mycobacterium*	56	*M. doricum*	5.74	*M. doricum*	0.45
*Nocardioides*	43	*N. marinisabuli*	5.10	*N. marinisabuli*	1.23
*Pseudomonas*	291	*P. stutzeri*	8.38	*P. aeruginosa*	5.12
*Rickettsia*	4	*R. prowazekii*	0.060	*R. prowazekii*	0.01
*Salmonella*	2	*S. enterica*	5.69	*S. enterica*	0.13
*Shigella*	4	*S. flexneri* *S. sonnei*	0.49 0.02	*S. boydii* *S. sonnei*	0.006 NR
*Staphylococcus*	36	*S. aureus* *S. delphini*	0.14 11.5	*S. aureus* *S. hominis*	5.54 24.3
*Vibrio*	80	*V. alginolyticus* *V. cholerae* *V. fluvialis* *V. parahaemolyticus* *V. vulnificus*	110000 9300 4800 15000 2900	*V. alginolyticus* *V. cholerae* *V. fluvialis* *V. parahaemolyticus* *V. vulnificus*	2000 190 23.4 180 26.3

Abbreviation: NR, no reads.

## Data Availability

The data that support the findings of this study are openly available in Sequence Read Archive (SRA) at https://www.ncbi.nlm.nih.gov/sra/docs/, Reference Number PRJNA1093391.

## Footnotes

1
^∗^Correction added on 17 October, after first online publication: Author.

2
^∗^Correction added on 17 October 2025, after first online publication: Helena M. Solo-Gabriele has been added as second corresponding author.

## Supporting Information

Supporting Information Additional supporting information can be found online in the Supporting Information section. Details about sequencing results, including abundances within bacterial groups targeted within this study.
