# Correction to “Use of Deep Sequencing to Evaluate Transitions in Microbial Communities in Stranded *Sargassum*”

**DOI:** 10.1155/ijm/9821430

**Published:** 2025-11-10

A. A. Abdool-Ghany, K. M. Babler, D. Bogumil, et al., “Use of Deep Sequencing to Evaluate Transitions in Microbial Communities in Stranded *Sargassum*,” *International Journal of Microbiology*, 2025 (2025): 3915271, 11 pages, https://doi.org/10.1155/ijm/3915271.

In the article titled “Use of Deep Sequencing to Evaluate Transitions in Microbial Communities in Stranded *Sargassum*,” author Helena M. Solo-Gabriele should have been added as a second corresponding author.

The online version of this article has been corrected accordingly.

We apologize for this error.

