# Supplementary material for: Use of Deep Sequencing to Evaluate Transitions in Microbial Communities in Stranded Sargassum
Source: Int J Microbiol. 2025 Jul 21;2025:3915271. doi: 10.1155/ijm/3915271 (PMC12303635; doi:10.1155/ijm/3915271)
Supplement: Supporting Information — Additional supporting information can be found online in the Supporting Information section. Details about sequencing results, including abundances within bacterial groups targeted within this study. [file 3915271.f1.docx]

Supplemental Text For:

Use of Deep Sequencing to Evaluate Transitions in Microbial Communities in Stranded Sargassum

Afeefa A. Abdool-Ghany^1, 2, 3 *^, Kristina M. Babler^1, +^, David Bogumil^4^, Sarah Pollock^4^,

Jiayu Li^5^, Schonna R. Manning^3^, Helena M. Solo-Gabriele^1^

^1^Department of Chemical, Environmental, and Materials Engineering, University of Miami, Coral Gables, Florida, USA

^2^ Department of Planning and Analytics, Brizaga Inc., Fort Lauderdale, Florida, USA

^3^Department of Biological Sciences, Institute of Environment, Florida International University, North Miami, Florida, USA

^4^Department of Sequencing Operations, Ultima Genomics Inc., Fremont, California , USA

^5^Department of Mechanical and Aerospace Engineering, University of Miami, Coral Gables, Florida, USA

For consideration for potential publication in: *International Journal of Microbiology*

Version Date: May 26, 2025

*Corresponding Author. Tel. +1-954-298-4073, Email address: aaa625@miami.edu. Department of Chemical, Environmental, and Materials Engineering, University of Miami, Coral Gables, Florida, USA

^+^ Currently with the Department of Human Genetics, University of Utah, Salt Lake City, Utah , USA

Table S-1: Number of Reads per extract

|  | Extract Number | Number of reads |
| --- | --- | --- |
| STS | 1b | 3730268 |
|  | 2a | 4861067 |
|  | 2b | 4077957 |
|  | 3b | 4120431 |
|  | 4a | 4214751 |
|  | 4b | 3442628 |
|  | 5a | 3304692 |
|  | 5b | 4212492 |
|  | 6b | 5849902 |
| LTS | 1a | 14102355 |
|  | 1b | 15496247 |
|  | 2a | 15828601 |
|  | 2b | 11696316 |
|  | 3a | 14215687 |
|  | 3b | 15650384 |
|  | 4a | 13697308 |
|  | 4b | 17634734 |
|  | 5a | 16135630 |
|  | 5b | 13168061 |
|  | 6a | 18501570 |

|  | Sample ID | dsDNA Concentration (ng/uL) | Sequencing pass/fail |
| --- | --- | --- | --- |
| STS | 1a | 12.235 | Fail |
|  | 1b | 15.350 | Pass |
|  | 2a | 12.691 | Pass |
|  | 2b | 14.984 | Pass |
|  | 3a | 13.289 | Fail |
|  | 3b | 10.922 | Pass |
|  | 4a | 12.692 | Pass |
|  | 4b | 11.247 | Pass |
|  | 5a | 14.973 | Pass |
|  | 5b | 11.251 | Pass |
|  | 6a | 13.035 | Pass |
|  | 6b | 11.156 | Fail |
| LTS | 1a | 38.888 | Pass |
|  | 1b | 39.109 | Pass |
|  | 2a | 36.854 | Pass |
|  | 2b | 41.776 | Pass |
|  | 3a | 32.788 | Pass |
|  | 3b | 36.824 | Pass |
|  | 4a | 20.187 | Pass |
|  | 4b | 19.353 | Pass |
|  | 5a | 17.818 | Pass |
|  | 5b | 19.121 | Pass |
|  | 6a | 16.748 | Pass |
|  | 6b | 17.084 | Fail |

Table S-2: dsDNA concentrations and Tape Station results

**Gut Commensals**

Table S-3: *Bacteroides* species detected.

| **Species Name** | **STS Average (ppm)** | **LTS Average (ppm)** |
| --- | --- | --- |
| *Bacteroides caccae* | 2.335 | 0.152 |
| *Bacteroides caecimuris* | 0.059 | 0.006 |
| *Bacteroides cellulosilyticus* | 0.914 | 0.096 |
| *Bacteroides eggerthii* | 1.237 | 0.093 |
| *Bacteroides faecis* | 0.435 | 0.049 |
| *Bacteroides fragilis* | 1.919 | 0.103 |
| *Bacteroides heparinolyticus* | 0.027 | 0.012 |
| *Bacteroides intestinalis* | 0.438 | 0.074 |
| *Bacteroides luhongzhouii* | 0.355 | 0.005 |
| *Bacteroides nordii* | 0.199 | 0.018 |
| *Bacteroides ovatus* | 2.828 | 0.117 |
| *Bacteroides salyersiae* | 0.499 | 0.056 |
| *Bacteroides* sp. A1C1 | 0.060 | 0.011 |
| *Bacteroides* sp. CACC 737 | 0.144 | 0.015 |
| *Bacteroides* sp. CBA7301 | 0.056 | 0.052 |
| *Bacteroides* sp. HF-162 | 0.060 | 0.000 |
| *Bacteroides* sp. KGMB07931 | 0.026 | 0.006 |
| *Bacteroides* sp. M10 | 0.145 | 0.007 |
| *Bacteroides* sp. PHL 2737 | 0.000 | 0.008 |
| *Bacteroides stercoris* | 3.786 | 0.214 |
| *Bacteroides thetaiotaomicron* | 3.195 | 0.171 |
| *Bacteroides uniformis* | 4.647 | 0.320 |
| *Bacteroides xylanisolvens* | 2.272 | 0.084 |
| *Bacteroides zhangwenhongii* | 0.239 | 0.018 |
| *Bacteroides zoogleoformans* | 0.000 | 0.035 |

Table S-4: *Bifidobacterium* species detected.

| **Species Name** | **STS Average (ppm)** | **LTS Average (ppm)** |
| --- | --- | --- |
| *Bifidobacterium adolescentis* | 2.807 | 0.112 |
| *Bifidobacterium angulatum* | 0.027 | 0.007 |
| *Bifidobacterium animalis* | 0.109 | 0.025 |
| *Bifidobacterium asteroides* | 0.290 | 0.021 |
| *Bifidobacterium bifidum* | 0.783 | 0.051 |
| *Bifidobacterium breve* | 0.057 | 0.078 |
| *Bifidobacterium catenulatum* | 0.260 | 0.022 |
| *Bifidobacterium choerinum* | 0.053 | 0.000 |
| *Bifidobacterium coryneforme* | 0.000 | 0.000 |
| *Bifidobacterium dentium* | 0.053 | 0.026 |
| *Bifidobacterium eulemuris* | 0.034 | 0.000 |
| *Bifidobacterium lemurum* | 0.026 | 0.000 |
| *Bifidobacterium longum* | 1.564 | 0.107 |
| *Bifidobacterium pseudocatenulatum* | 0.794 | 0.063 |
| *Bifidobacterium pseudolongum* | 0.087 | 0.000 |
| *Bifidobacterium saguini* | 0.000 | 0.007 |
| *Bifidobacterium subtile* | 0.057 | 0.006 |
| *Bifidobacterium thermophilum* | 0.707 | 0.032 |

Table S-5: *Clostridium* species detected.

| **Species Name** | **STS Average (ppm)** | **LTS Average (ppm)** |
| --- | --- | --- |
| *Clostridium aceticum* | 0.027 | 0.012 |
| *Clostridium argentinense* | 0.034 | 0.000 |
| *Clostridium baratii* | 0.026 | 0.007 |
| *Clostridium beijerinckii* | 0.099 | 0.007 |
| *Clostridium bornimense* | 0.000 | 1.927 |
| *Clostridium botulinum* | 0.141 | 0.040 |
| *Clostridium butyricum* | 0.077 | 0.037 |
| *Clostridium cadaveris* | 0.032 | 0.000 |
| *Clostridium chauvoei* | 0.027 | 0.000 |
| *Clostridium cochlearium* | 0.000 | 0.368 |
| *Clostridium diolis* | 0.000 | 0.008 |
| *Clostridium fermenticellae* | 0.032 | 0.000 |
| *Clostridium formicaceticum* | 0.000 | 0.019 |
| *Clostridium gasigenes* | 0.000 | 0.012 |
| *Clostridium intestinale* | 1.984 | 0.174 |
| *Clostridium isatidis* | 0.000 | 0.042 |
| *Clostridium kluyveri* | 0.056 | 2.509 |
| *Clostridium manihotivorum* | 0.042 | 0.012 |
| *Clostridium perfringens* | 0.228 | 0.036 |
| *Clostridium saccharobutylicum* | 0.046 | 0.007 |
| *Clostridium saccharoperbutylacetonicum* | 0.000 | 0.011 |
| *Clostridium scatologenes* | 0.060 | 0.005 |
| *Clostridium septicum* | 0.000 | 0.005 |
| *Clostridium* sp. 001 | 0.000 | 0.007 |
| *Clostridium* sp. BNL1100 | 0.030 | 0.005 |
| *Clostridium* sp. C5S11 | 0.084 | 0.013 |
| *Clostridium* sp. 'deep sea' | 0.091 | 0.019 |
| *Clostridium* sp. DL-VIII | 0.107 | 0.029 |
| *Clostridium* sp. JN-1 | 0.023 | 0.000 |
| *Clostridium* sp. JN-9 | 0.053 | 0.006 |
| *Clostridium* sp. M62/1 | 0.335 | 0.030 |
| *Clostridium* sp. SY8519 | 0.019 | 0.005 |
| *Clostridium sporogenes* | 0.057 | 0.000 |
| *Clostridium tetani* | 0.023 | 0.000 |
| *Clostridium thermarum* | 0.000 | 0.939 |

Table S-6: *Eubacterium* species detected.

| **Species Name** | **STS Average (ppm)** | **LTS Average (ppm)** |
| --- | --- | --- |
| *Eubacterium callanderi* | 0.032 | 0.012 |
| *Eubacterium limosum* | 0.030 | 0.006 |
| *Eubacterium* sp. MSJ-33 | 0.032 | 0.018 |
| *Eubacterium* sp. NSJ-61 | 0.045 | 0.000 |

Table S-7: *Enterococcus* species detected.

| **Species Name** | **STS Average (ppm)** | **LTS Average (ppm)** |
| --- | --- | --- |
| *Enterococcus avium* | 0.057 | 0.057 |
| *Enterococcus casseliflavus* | 0.079 | 0.006 |
| *Enterococcus cecorum* | 0.136 | 0.030 |
| *Enterococcus durans* | 0.086 | 0.018 |
| *Enterococcus faecalis* | 0.950 | 0.418 |
| *Enterococcus faecium* | 0.210 | 0.066 |
| *Enterococcus gallinarum* | 0.000 | 0.075 |
| *Enterococcus gilvus* | 0.000 | 0.021 |
| *Enterococcus hirae* | 0.134 | 1.605 |
| *Enterococcus innesii* | 0.000 | 0.006 |
| *Enterococcus mundtii* | 0.121 | 0.006 |
| *Enterococcus raffinosus* | 0.034 | 0.026 |
| *Enterococcus saigonensis* | 0.097 | 0.023 |
| *Enterococcus thailandicus* | 0.032 | 0.024 |
| *Enterococcus wangshanyuanii* | 0.055 | 0.012 |

Table S-8: *Lactobacillus* species detected.

| **Species Name** | **STS Average (ppm)** | **LTS Average (ppm)** |
| --- | --- | --- |
| *Lactobacillus acetotolerans* | 0.318 | 0.007 |
| *Lactobacillus acidophilus* | 0.032 | 0.000 |
| *Lactobacillus amylolyticus* | 0.000 | 0.007 |
| *Lactobacillus amylovorus* | 0.030 | 0.028 |
| *Lactobacillus apis* | 0.101 | 0.006 |
| *Lactobacillus crispatus* | 2.078 | 0.112 |
| *Lactobacillus delbrueckii* | 0.754 | 0.074 |
| *Lactobacillus gasseri* | 0.087 | 0.006 |
| *Lactobacillus helsingborgensis* | 0.019 | 0.000 |
| *Lactobacillus iners* | 0.481 | 0.047 |
| *Lactobacillus jensenii* | 0.278 | 0.014 |
| *Lactobacillus johnsonii* | 0.108 | 0.019 |
| *Lactobacillus kefiranofaciens* | 0.000 | 0.006 |
| *Lactobacillus kullabergensis* | 0.114 | 0.005 |
| *Lactobacillus sp. 3B(2020)* | 0.026 | 0.000 |
| *Lactobacillus sp. CBA3605* | 0.027 | 0.013 |
| *Lactobacillus taiwanensis* | 0.045 | 0.006 |
| *Lactobacillus ultunensis* | 0.030 | 0.000 |

**Fecal Coliforms**

Table S-9: *Citrobacter* species detected.

| **Species Name** | **STS Average (ppm)** | **LTS Average (ppm)** |
| --- | --- | --- |
| *Citrobacter amalonaticus* | 0.000 | 0.005 |
| *Citrobacter arsenatis* | 0.188 | 0.000 |
| *Citrobacter braakii* | 0.000 | 0.289 |
| *Citrobacter cronae* | 0.219 | 0.000 |
| *Citrobacter farmeri* | 1.731 | 0.157 |
| *Citrobacter freundii* | 9.108 | 0.150 |
| *Citrobacter freundii complex sp. CFNIH2* | 0.246 | 0.007 |
| *Citrobacter freundii complex sp. CFNIH3* | 0.027 | 0.000 |
| *Citrobacter koseri* | 0.439 | 0.037 |
| *Citrobacter pasteurii* | 0.475 | 0.073 |
| *Citrobacter portucalensis* | 0.305 | 0.024 |
| *Citrobacter rodentium* | 0.916 | 0.112 |
| *Citrobacter sedlakii* | 0.000 | 0.079 |
| *Citrobacter sp. BDA59-3* | 0.809 | 0.018 |
| *Citrobacter sp. BIDMC107* | 0.225 | 0.000 |
| *Citrobacter sp. CF971* | 0.059 | 0.000 |
| *Citrobacter sp. CRE-46* | 0.000 | 0.012 |
| *Citrobacter sp. MGH 55* | 0.067 | 0.000 |
| *Citrobacter sp. R56* | 1.786 | 0.139 |
| *Citrobacter sp. RHB25-C09* | 1.036 | 0.092 |
| *Citrobacter sp. RHBSTW-00535* | 0.032 | 0.000 |
| *Citrobacter sp. Y3* | 0.089 | 0.000 |
| *Citrobacter tructae* | 1.029 | 0.007 |
| *Citrobacter werkmanii* | 0.336 | 0.067 |
| *Citrobacter youngae* | 0.563 | 0.019 |

Table S-10: *Enterobacter* species detected.

| **Species Name** | **STS Average (ppm)** | **LTS Average (ppm)** |
| --- | --- | --- |
| *Enterobacter asburiae* | 3.978 | 0.117 |
| *Enterobacter bugandensis* | 0.157 | 0.014 |
| *Enterobacter cancerogenus* | 0.180 | 0.028 |
| *Enterobacter chengduensis* | 0.060 | 0.057 |
| *Enterobacter chuandaensis* | 0.534 | 0.013 |
| *Enterobacter cloacae* | 3.439 | 0.212 |
| *Enterobacter cloacae* complex sp. | 0.030 | 0.025 |
| *Enterobacter cloacae* complex sp. FDA-CDC-AR_0132 | 0.452 | 0.000 |
| *Enterobacter cloacae* complex sp. FDA-CDC-AR_0164 | 0.060 | 0.000 |
| *Enterobacter hormaechei* | 6.423 | 1.096 |
| *Enterobacter huaxiensis* | 2.942 | 0.019 |
| *Enterobacter kobei* | 1.164 | 1.453 |
| *Enterobacter ludwigii* | 1.098 | 0.036 |
| *Enterobacter mori* | 0.187 | 0.042 |
| *Enterobacter oligotrophicus* | 0.190 | 0.007 |
| *Enterobacter roggenkampii* | 5.727 | 0.038 |
| *Enterobacter sichuanensis* | 0.050 | 0.032 |
| *Enterobacter soli* | 0.542 | 0.000 |
| *Enterobacter* sp. BIDMC 29 | 0.393 | 0.013 |
| *Enterobacter* sp. BIDMC100 | 0.000 | 0.012 |
| *Enterobacter* sp. Colony194 | 0.109 | 0.000 |
| *Enterobacter* sp. E76 | 0.440 | 0.051 |
| *Enterobacter* sp. HK169 | 0.000 | 0.005 |
| *Enterobacter* sp. JBIWA003 | 0.413 | 0.000 |
| *Enterobacter* sp. JBIWA005 | 0.000 | 0.006 |
| *Enterobacter* sp. JUb54 | 1.297 | 0.131 |
| *Enterobacter* sp. LU1 | 0.100 | 0.000 |
| *Enterobacter* sp. N18-03635 | 0.030 | 0.026 |
| *Enterobacter* sp. RHBSTW-00175 | 0.196 | 0.000 |
| *Enterobacter* sp. RHBSTW-00975 | 0.641 | 0.006 |
| *Enterobacter* sp. RHBSTW-00994 | 0.356 | 0.126 |
| *Enterobacter* sp. SA187 | 0.947 | 0.043 |
| *Enterobacter* sp. SES19 | 0.023 | 0.005 |
| *Enterobacter* sp. SGAir0187 | 0.066 | 0.000 |

Table S-11: *Escherichia* species detected.

| **Species Name** | **STS Average (ppm)** | **LTS Average (ppm)** |
| --- | --- | --- |
| *Escherichia albertii* | 0.546 | 0.038 |
| *Escherichia coli* | 40.221 | 1.487 |
| *Escherichia fergusonii* | 4.389 | 0.014 |
| *Escherichia marmotae* | 1.671 | 0.044 |
| *Escherichia sp. E4742* | 0.108 | 0.006 |

Table S-12: *Klebsiella* species detected.

| **Species Name** | **STS Average (ppm)** | **LTS Average (ppm)** |
| --- | --- | --- |
| *Klebsiella aerogenes* | 0.604 | 0.093 |
| *Klebsiella africana* | 0.135 | 0.044 |
| *Klebsiella grimontii* | 0.155 | 0.014 |
| *Klebsiella huaxiensis* | 0.050 | 0.030 |
| *Klebsiella michiganensis* | 7.466 | 0.193 |
| *Klebsiella oxytoca* | 1.065 | 0.165 |
| *Klebsiella pneumoniae* | 140.487 | 11.808 |
| *Klebsiella quasipneumoniae* | 1.777 | 0.497 |
| *Klebsiella quasivariicola* | 0.054 | 0.012 |
| *Klebsiella* sp. A52 | 0.000 | 0.005 |
| *Klebsiella* sp. BDA134-6 | 0.046 | 0.000 |
| *Klebsiella* sp. LTGPAF-6F | 0.000 | 0.006 |
| *Klebsiella* sp. P1CD1 | 0.026 | 0.000 |
| *Klebsiella* sp. WP4-W18-ESBL-05 | 0.030 | 0.000 |
| *Klebsiella variicola* | 0.750 | 0.029 |

**Pathogenic Genus**

Table S-13: *Campylobacter* species detected.

| **Species Name** | **STS Average (ppm)** | **LTS Average (ppm)** |
| --- | --- | --- |
| *Campylobacter blaseri* | 0.000 | 0.017 |
| *Campylobacter canadensis* | 0.059 | 0.000 |
| *Campylobacter coli* | 0.057 | 0.011 |
| *Campylobacter concisus* | 1.367 | 0.053 |
| *Campylobacter cuniculorum* | 0.034 | 0.000 |
| *Campylobacter fetus* | 0.000 | 0.006 |
| *Campylobacter geochelonis* | 0.000 | 0.006 |
| *Campylobacter gracilis* | 0.000 | 0.006 |
| *Campylobacter hepaticus* | 0.054 | 0.000 |
| *Campylobacter hominis* | 0.030 | 0.000 |
| *Campylobacter iguaniorum* | 0.030 | 0.000 |
| *Campylobacter insulaenigrae* | 0.023 | 0.000 |
| *Campylobacter jejuni* | 0.243 | 0.000 |
| *Campylobacter lanienae* | 0.026 | 0.000 |
| *Campylobacter mucosalis* | 0.000 | 0.006 |
| *Campylobacter peloridis* | 0.095 | 0.000 |
| *Campylobacter showae* | 0.053 | 0.000 |
| *Campylobacter* sp. 19-13652 | 0.023 | 0.000 |
| *Campylobacter* sp. CCUG 57310 | 0.000 | 0.012 |
| *Campylobacter* sp. RM10537 | 0.026 | 0.000 |
| *Campylobacter* sp. RM12651 | 0.057 | 0.000 |
| *Campylobacter* sp. RM5004 | 0.027 | 0.000 |
| *Campylobacter sputorum* | 0.079 | 0.000 |
| *Campylobacter subantarcticus* | 0.023 | 0.000 |
| *Campylobacter volucris* | 0.054 | 0.000 |

Table S-14: *Corynebacterium* species detected.

| **Species Name** | **STS Average (ppm)** | **LTS Average (ppm)** |
| --- | --- | --- |
| *Corynebacterium ammoniagenes* | 0.053 | 0.018 |
| *Corynebacterium amycolatum* | 0.086 | 0.000 |
| *Corynebacterium anserum* | 0.105 | 0.012 |
| *Corynebacterium atypicum* | 0.027 | 0.012 |
| *Corynebacterium aurimucosum* | 0.488 | 0.099 |
| *Corynebacterium camporealensis* | 0.054 | 0.038 |
| *Corynebacterium comes* | 0.000 | 0.018 |
| *Corynebacterium coyleae* | 0.319 | 0.041 |
| *Corynebacterium crudilactis* | 0.053 | 0.008 |
| *Corynebacterium diphtheriae* | 0.119 | 0.033 |
| *Corynebacterium epidermidicanis* | 0.026 | 0.006 |
| *Corynebacterium falsenii* | 0.000 | 0.034 |
| *Corynebacterium flavescens* | 0.479 | 0.025 |
| *Corynebacterium freneyi* | 0.164 | 0.046 |
| *Corynebacterium gerontici* | 0.054 | 0.000 |
| *Corynebacterium glaucum* | 0.026 | 0.000 |
| *Corynebacterium glucuronolyticum* | 0.000 | 0.005 |
| *Corynebacterium glutamicum* | 0.076 | 0.007 |
| *Corynebacterium imitans* | 0.215 | 0.006 |
| *Corynebacterium incognita* | 0.126 | 0.006 |
| *Corynebacterium jeikeium* | 1.080 | 0.072 |
| *Corynebacterium kalinowskii* | 0.249 | 0.084 |
| *Corynebacterium kefirresidentii* | 0.628 | 0.078 |
| *Corynebacterium kroppenstedtii* | 0.284 | 0.036 |
| *Corynebacterium liangguodongii* | 0.089 | 0.000 |
| *Corynebacterium lizhenjunii* | 0.129 | 0.035 |
| *Corynebacterium lujinxingii* | 0.195 | 0.000 |
| *Corynebacterium macginleyi* | 0.057 | 0.005 |
| *Corynebacterium matruchotii* | 0.455 | 0.050 |
| *Corynebacterium minutissimum* | 0.167 | 0.042 |
| *Corynebacterium mustelae* | 0.102 | 0.000 |
| *Corynebacterium mycetoides* | 0.027 | 0.018 |
| *Corynebacterium occultum* | 0.027 | 0.018 |
| *Corynebacterium pelargi* | 0.030 | 0.000 |
| *Corynebacterium propinquum* | 0.108 | 0.030 |
| *Corynebacterium provencense* | 0.134 | 0.000 |
| *Corynebacterium pseudopelargi* | 0.000 | 0.005 |
| *Corynebacterium pseudotuberculosis* | 0.060 | 0.013 |
| *Corynebacterium qintianiae* | 0.000 | 0.100 |
| *Corynebacterium renale* | 0.109 | 0.000 |
| *Corynebacterium riegelii* | 0.205 | 0.532 |
| *Corynebacterium sanguinis* | 0.152 | 0.043 |
| *Corynebacterium segmentosum* | 0.057 | 0.107 |
| *Corynebacterium silvaticum* | 0.080 | 0.020 |
| *Corynebacterium simulans* | 0.162 | 0.026 |
| *Corynebacterium singulare* | 0.212 | 0.007 |
| *Corynebacterium* sp. 1864 | 0.027 | 0.006 |
| *Corynebacterium* sp. 4H37-19 | 0.000 | 0.005 |
| *Corynebacterium* sp. CNCTC7651 | 0.095 | 0.007 |
| *Corynebacterium* sp. FDAARGOS 1242 | 0.055 | 0.006 |
| *Corynebacterium sp. NML98-0116* | 0.185 | 0.012 |
| *Corynebacterium* sp. sy039 | 0.026 | 0.006 |
| *Corynebacterium stationis* | 1.194 | 0.884 |
| *Corynebacterium striatum* | 0.350 | 0.244 |
| *Corynebacterium suranareeae* | 0.027 | 0.005 |
| *Corynebacterium testudinoris* | 0.034 | 0.000 |
| *Corynebacterium timonense* | 0.032 | 0.037 |
| *Corynebacterium tuberculostearicum* | 5.366 | 0.463 |
| *Corynebacterium uberis* | 0.179 | 0.006 |
| *Corynebacterium ulcerans* | 0.046 | 0.000 |
| *Corynebacterium urealyticum* | 0.111 | 0.058 |
| *Corynebacterium ureicelerivorans* | 0.279 | 0.181 |
| *Corynebacterium urogenitale* | 0.000 | 0.030 |
| *Corynebacterium uterequi* | 0.000 | 0.029 |
| *Corynebacterium xerosis* | 0.151 | 0.037 |
| *Corynebacterium yudongzhengii* | 0.000 | 0.006 |
| *Corynebacterium zhongnanshanii* | 0.393 | 0.012 |

Table S-15: *Fictibacillus* species detected.

| **Species Name** | **STS Average (ppm)** | **LTS Average (ppm)** |
| --- | --- | --- |
| *Fictibacillus arsenicus* | 0.023 | 0.125 |
| *Fictibacillus phosphorivorans* | 0.341 | 0.254 |

Table S-16: *Francisella* species detected.

| **Species Name** | **STS Average (ppm)** | **LTS Average (ppm)** |
| --- | --- | --- |
| *Francisella adeliensis* | 0.192 | 0.000 |
| *Francisella frigiditurris* | 0.557 | 0.000 |
| *Francisella halioticida* | 0.911 | 0.000 |
| *Francisella hispaniensis* | 0.810 | 0.000 |
| *Francisella marina* | 0.059 | 0.000 |
| *Francisella opportunistica* | 0.121 | 0.000 |
| *Francisella orientalis* | 0.148 | 0.006 |
| *Francisella philomiragia* | 3.944 | 0.011 |
| *Francisella salimarina* | 0.200 | 0.000 |
| *Francisella salina* | 0.279 | 0.000 |
| *Francisella* sp. FSC1006 | 0.317 | 0.012 |
| *Francisella* sp. LA112445 | 1.641 | 0.012 |
| *Francisella* sp. Scap27 | 0.087 | 0.000 |
| *Francisella tularensis* | 104.216 | 0.075 |
| *Francisella uliginis* | 39.956 | 0.040 |

Table S-17: *Legionella* species detected.

| **Species Name** | **STS Average (ppm)** | **LTS Average (ppm)** |
| --- | --- | --- |
| *Legionella adelaidensis* | 0.056 | 0.011 |
| *Legionella anisa* | 0.245 | 0.006 |
| *Legionella antarctica* | 0.104 | 0.027 |
| *Legionella cherrii* | 0.027 | 0.025 |
| *Legionella clemsonensis* | 0.198 | 0.012 |
| *Legionella* endosymbiont of Polyplax serrata | 0.027 | 0.000 |
| *Legionella geestiana* | 0.079 | 0.085 |
| *Legionella hackeliae* | 0.126 | 0.017 |
| *Legionella israelensis* | 0.336 | 0.428 |
| *Legionella jordanis* | 0.296 | 0.071 |
| *Legionella lansingensis* | 0.084 | 0.023 |
| *Legionella longbeachae* | 0.207 | 0.013 |
| *Legionella pneumophila* | 1.058 | 0.029 |
| *Legionella sainthelensi* | 2.010 | 0.006 |
| *Legionella* sp. MW5194 | 0.730 | 0.178 |
| *Legionella* sp. PC1000 | 0.213 | 0.000 |
| *Legionella* sp. PC997 | 0.493 | 0.000 |
| *Legionella spiritensis* | 0.329 | 0.181 |
| *Legionella waltersii* | 0.065 | 0.023 |

Table S-18: *Listeria* species detected.

| **Species Name** | **STS Average (ppm)** | **LTS Average (ppm)** |
| --- | --- | --- |
| *Listeria grayi* | 0.189 | 0.032 |
| *Listeria innocua* | 0.000 | 0.225 |
| *Listeria ivanovii* | 0.000 | 0.081 |
| *Listeria monocytogenes* | 0.241 | 1.213 |
| *Listeria seeligeri* | 0.000 | 0.045 |
| *Listeria* sp. PSOL-1 | 0.000 | 0.014 |
| *Listeria weihenstephanensis* | 0.000 | 0.065 |
| *Listeria welshimeri* | 0.081 | 0.021 |

Table S-19: *Mycobacterium* species detected.

| **Species Name** | **STS Average (ppm)** | **LTS Average (ppm)** |
| --- | --- | --- |
| *Mycobacterium avium* | 0.027 | 0.012 |
| *Mycobacterium basiliense* | 0.287 | 0.020 |
| *Mycobacterium conspicuum* | 0.000 | 0.013 |
| *Mycobacterium cookii* | 0.000 | 0.047 |
| *Mycobacterium diernhoferi* | 0.060 | 0.006 |
| *Mycobacterium dioxanotrophicus* | 0.219 | 0.063 |
| *Mycobacterium doricum* | 5.735 | 0.449 |
| *Mycobacterium florentinum* | 0.000 | 0.006 |
| *Mycobacterium frederiksbergense* | 0.091 | 0.007 |
| *Mycobacterium goodii* | 0.544 | 0.012 |
| *Mycobacterium gordonae* | 0.000 | 0.020 |
| *Mycobacterium grossiae* | 0.151 | 0.057 |
| *Mycobacterium heckeshornense* | 0.026 | 0.040 |
| *Mycobacterium heidelbergense* | 0.049 | 0.013 |
| *Mycobacterium holsaticum* | 0.057 | 0.029 |
| *Mycobacterium intracellulare* | 0.019 | 0.012 |
| *Mycobacterium kansasii* | 0.127 | 0.013 |
| *Mycobacterium koreense* | 0.000 | 0.006 |
| *Mycobacterium kubicae* | 0.081 | 0.064 |
| *Mycobacterium lacus* | 0.000 | 0.006 |
| *Mycobacterium lentiflavum* | 0.108 | 0.000 |
| *Mycobacterium leprae* | 0.027 | 0.000 |
| *Mycobacterium lepromatosis* | 0.000 | 0.010 |
| *Mycobacterium malmoense* | 0.023 | 0.057 |
| *Mycobacterium mantenii* | 0.032 | 0.005 |
| *Mycobacterium marinum* | 0.023 | 0.000 |
| *Mycobacterium marseillense* | 0.049 | 0.000 |
| *Mycobacterium noviomagense* | 0.000 | 0.008 |
| *Mycobacterium ostraviense* | 0.000 | 0.000 |
| *Mycobacterium pallens* | 0.032 | 0.012 |
| *Mycobacterium paragordonae* | 0.059 | 0.000 |
| *Mycobacterium paraseoulense* | 0.000 | 0.038 |
| *Mycobacterium paraterrae* | 0.000 | 0.038 |
| *Mycobacterium riyadhense* | 0.000 | 0.006 |
| *Mycobacterium rufum* | 0.000 | 0.007 |
| *Mycobacterium saskatchewanense* | 0.114 | 0.006 |
| *Mycobacterium senegalense* | 0.030 | 0.000 |
| *Mycobacterium seoulense* | 0.000 | 0.012 |
| *Mycobacterium shigaense* | 0.059 | 0.018 |
| *Mycobacterium shinjukuense* | 0.000 | 0.006 |
| *Mycobacterium simiae* | 0.164 | 0.038 |
| *Mycobacterium* sp. DL440 | 0.027 | 0.000 |
| *Mycobacterium* sp. DL592 | 0.089 | 0.006 |
| *Mycobacterium* sp. EPa45 | 0.030 | 0.062 |
| *Mycobacterium* sp. IDR2000157661 | 0.027 | 0.007 |
| *Mycobacterium* sp. JS623 | 0.000 | 0.011 |
| *Mycobacterium* sp. MS1601 | 0.000 | 0.024 |
| *Mycobacterium* sp. TY59 | 0.120 | 0.046 |
| *Mycobacterium* sp. WY10 | 0.000 | 0.012 |
| *Mycobacterium* sp. YC-RL4 | 0.034 | 0.000 |
| *Mycobacterium spongiae* | 0.050 | 0.025 |
| *Mycobacterium stomatepiae* | 0.105 | 0.000 |
| *Mycobacterium vicinigordonae* | 0.000 | 0.012 |
| *Mycobacterium virginiense* | 0.108 | 0.000 |
| *Mycobacterium xenopi* | 0.388 | 0.012 |

Table S-20: *Nocardioides* species detected.

| **Species Name** | **STS Average (ppm)** | **LTS Average (ppm)** |
| --- | --- | --- |
| *Nocardioides anomalus* | 0.079 | 0.025 |
| *Nocardioides aquaticus* | 0.140 | 0.025 |
| *Nocardioides aromaticivorans* | 0.191 | 0.079 |
| *Nocardioides baekrokdamisoli* | 0.072 | 0.005 |
| *Nocardioides cynanchi* | 0.382 | 0.073 |
| *Nocardioides daphniae* | 0.443 | 0.130 |
| *Nocardioides dongkuii* | 0.222 | 0.056 |
| *Nocardioides euryhalodurans* | 0.107 | 0.047 |
| *Nocardioides houyundeii* | 0.094 | 0.020 |
| *Nocardioides humi* | 0.350 | 0.097 |
| *Nocardioides jishulii* | 0.000 | 0.038 |
| *Nocardioides marinisabuli* | 5.099 | 1.228 |
| *Nocardioides mesophilus* | 0.132 | 0.033 |
| *Nocardioides okcheonensis* | 0.080 | 0.006 |
| *Nocardioides panacisoli* | 4.935 | 0.717 |
| *Nocardioides piscis* | 0.062 | 0.007 |
| *Nocardioides rotundus* | 2.685 | 0.083 |
| *Nocardioides sambongensis* | 0.094 | 0.031 |
| *Nocardioides seonyuensis* | 0.053 | 0.012 |
| *Nocardioides* sp. 603 | 0.054 | 0.006 |
| *Nocardioides* sp. CF8 | 0.212 | 0.006 |
| *Nocardioides* sp. cx-173 | 0.110 | 0.044 |
| *Nocardioides* sp. dk884 | 0.000 | 0.011 |
| *Nocardioides* sp. G188 | 0.086 | 0.028 |
| *Nocardioides* sp. HDW12B | 1.403 | 0.216 |
| *Nocardioides* sp. InS609-2 | 0.057 | 0.066 |
| *Nocardioides* sp. JQ2195 | 0.054 | 0.010 |
| *Nocardioides* sp. JS614 | 0.223 | 0.023 |
| *Nocardioides* sp. Kera G14 | 0.027 | 0.006 |
| *Nocardioides* sp. LMS-CY | 0.026 | 0.061 |
| *Nocardioides* sp. MC1495 | 0.091 | 0.043 |
| *Nocardioides* sp. S-1144 | 0.080 | 0.023 |
| *Nocardioides* sp. S5 | 0.393 | 0.034 |
| *Nocardioides* sp. SCSIO 67246 | 4.846 | 0.704 |
| *Nocardioides* sp. TF02-7 | 0.192 | 0.019 |
| *Nocardioides* sp. W7 | 0.056 | 0.024 |
| *Nocardioides* sp. WS12 | 0.212 | 0.015 |
| *Nocardioides* sp. zg-1228 | 0.032 | 0.053 |
| *Nocardioides* sp. zg-536 | 0.081 | 0.006 |
| *Nocardioides* sp. zg-579 | 0.026 | 0.005 |
| *Nocardioides* sp. ZJ1313 | 0.050 | 0.015 |
| *Nocardioides ungokensis* | 0.197 | 0.017 |
| *Nocardioides yefusunii* | 0.061 | 0.000 |

Table S-21: *Pseudomonas* species detected.

| **Species Name** | **STS Average (ppm)** | **LTS Average (ppm)** |
| --- | --- | --- |
| *Pseudomonas aeruginosa* | 7.293 | 5.124 |
| *Pseudomonas agarici* | 0.143 | 0.188 |
| *Pseudomonas alcaligenes* | 4.349 | 0.605 |
| *Pseudomonas alkylphenolica* | 0.298 | 0.164 |
| *Pseudomonas allokribbensis* | 0.066 | 0.006 |
| *Pseudomonas alvandae* | 0.159 | 0.025 |
| *Pseudomonas amygdali* | 0.167 | 0.017 |
| *Pseudomonas antarctica* | 0.192 | 0.075 |
| *Pseudomonas anuradhapurensis* | 0.000 | 0.163 |
| *Pseudomonas argentinensis* | 0.274 | 0.163 |
| *Pseudomonas arsenicoxydans* | 0.213 | 0.005 |
| *Pseudomonas asgharzadehiana* | 0.143 | 0.048 |
| *Pseudomonas asplenii* | 0.072 | 0.019 |
| *Pseudomonas asturiensis* | 0.428 | 0.181 |
| *Pseudomonas atacamensis* | 0.065 | 0.007 |
| *Pseudomonas avellanae* | 0.030 | 0.000 |
| *Pseudomonas azerbaijanoriens* | 0.084 | 0.057 |
| *Pseudomonas azotoformans* | 0.307 | 0.134 |
| *Pseudomonas balearica* | 3.128 | 0.517 |
| *Pseudomonas bijieensis* | 0.076 | 0.006 |
| *Pseudomonas brassicacearum* | 0.167 | 0.125 |
| *Pseudomonas brenneri* | 0.000 | 0.033 |
| *Pseudomonas campi* | 0.082 | 0.096 |
| *Pseudomonas cavernae* | 0.188 | 0.183 |
| *Pseudomonas cedrina* | 0.046 | 0.030 |
| *Pseudomonas cerasi* | 0.000 | 0.012 |
| *Pseudomonas chengduensis* | 0.073 | 0.109 |
| *Pseudomonas chloritidismutans* | 0.000 | 0.071 |
| *Pseudomonas chlororaphis* | 1.225 | 0.686 |
| *Pseudomonas cichorii* | 1.500 | 0.230 |
| *Pseudomonas citronellolis* | 0.471 | 0.281 |
| *Pseudomonas congelans* | 0.287 | 0.029 |
| *Pseudomonas coronafaciens* | 0.196 | 0.075 |
| *Pseudomonas corrugata* | 0.100 | 0.074 |
| *Pseudomonas cremoricolorata* | 0.703 | 0.222 |
| *Pseudomonas donghuensis* | 0.000 | 0.024 |
| *Pseudomonas entomophila* | 0.180 | 0.036 |
| *Pseudomonas eucalypticola* | 0.570 | 0.239 |
| *Pseudomonas extremaustralis* | 0.134 | 0.020 |
| *Pseudomonas fakonensis* | 0.000 | 0.037 |
| *Pseudomonas fluorescens* | 3.021 | 0.698 |
| *Pseudomonas fragi* | 0.274 | 0.147 |
| *Pseudomonas frederiksbergensis* | 0.393 | 0.266 |
| *Pseudomonas furukawaii* | 0.631 | 0.429 |
| *Pseudomonas fuscovaginae* | 0.166 | 0.019 |
| *Pseudomonas germanica* | 0.000 | 0.005 |
| *Pseudomonas glycinae* | 0.000 | 0.012 |
| *Pseudomonas gozinkensis* | 0.000 | 0.043 |
| *Pseudomonas graminis* | 0.153 | 0.072 |
| *Pseudomonas granadensis* | 0.059 | 0.017 |
| *Pseudomonas guangdongensis* | 1.058 | 0.301 |
| *Pseudomonas hamedanensis* | 0.205 | 0.045 |
| *Pseudomonas iranensis* | 0.060 | 0.074 |
| *Pseudomonas juntendi* | 0.118 | 0.023 |
| *Pseudomonas khazarica* | 0.027 | 0.038 |
| *Pseudomonas knackmussii* | 0.701 | 0.252 |
| *Pseudomonas koreensis* | 0.244 | 0.026 |
| *Pseudomonas kribbensis* | 0.030 | 0.034 |
| *Pseudomonas kunmingensis* | 0.000 | 0.206 |
| *Pseudomonas lactis* | 0.030 | 0.018 |
| *Pseudomonas lalkuanensis* | 0.128 | 0.319 |
| *Pseudomonas libanensis* | 0.000 | 0.255 |
| *Pseudomonas lini* | 0.084 | 0.295 |
| *Pseudomonas lundensis* | 2.772 | 0.684 |
| *Pseudomonas lurida* | 0.079 | 0.071 |
| *Pseudomonas luteola* | 0.000 | 0.058 |
| *Pseudomonas mandelii* | 0.000 | 0.006 |
| *Pseudomonas marincola* | 0.923 | 0.256 |
| *Pseudomonas maumuensis* | 0.027 | 0.111 |
| *Pseudomonas mediterranea* | 0.385 | 0.082 |
| *Pseudomonas mendocina* | 1.348 | 0.644 |
| *Pseudomonas migulae* | 0.056 | 0.030 |
| *Pseudomonas monsensis* | 0.027 | 0.006 |
| *Pseudomonas monteilii* | 0.510 | 0.216 |
| *Pseudomonas moraviensis* | 0.000 | 0.011 |
| *Pseudomonas mosselii* | 0.027 | 0.048 |
| *Pseudomonas mucidolens* | 0.332 | 0.207 |
| *Pseudomonas multiresinivorans* | 0.394 | 0.104 |
| *Pseudomonas muyukensis* | 0.019 | 0.040 |
| *Pseudomonas nanhaiensis* | 1.040 | 0.320 |
| *Pseudomonas nitroreducens* | 0.038 | 0.052 |
| *Pseudomonas ogarae* | 0.000 | 0.020 |
| *Pseudomonas oleovorans* | 0.557 | 0.114 |
| *Pseudomonas orientalis* | 0.526 | 0.204 |
| *Pseudomonas oryzae* | 1.029 | 0.277 |
| *Pseudomonas oryzihabitans* | 1.237 | 0.951 |
| *Pseudomonas oryziphila* | 0.149 | 0.014 |
| *Pseudomonas otitidis* | 0.510 | 0.476 |
| *Pseudomonas palleroniana* | 0.517 | 0.086 |
| *Pseudomonas parafulva* | 0.350 | 0.397 |
| *Pseudomonas phragmitis* | 0.891 | 1.199 |
| *Pseudomonas plecoglossicida* | 0.227 | 0.069 |
| *Pseudomonas poae* | 0.433 | 0.197 |
| *Pseudomonas pohangensis* | 0.367 | 0.644 |
| *Pseudomonas promysalinigenes* | 0.449 | 0.155 |
| *Pseudomonas prosekii* | 0.062 | 0.142 |
| *Pseudomonas protegens* | 0.278 | 0.787 |
| *Pseudomonas psychrophila* | 0.736 | 0.718 |
| *Pseudomonas psychrotolerans* | 1.387 | 0.820 |
| *Pseudomonas putida* | 3.994 | 3.714 |
| *Pseudomonas qingdaonensis* | 1.020 | 0.388 |
| *Pseudomonas reinekei* | 0.056 | 0.051 |
| *Pseudomonas rhizosphaerae* | 0.119 | 0.098 |
| *Pseudomonas rhodesiae* | 1.526 | 0.220 |
| *Pseudomonas salmasensis* | 0.026 | 0.000 |
| *Pseudomonas saudiphocaensis* | 0.420 | 0.286 |
| *Pseudomonas savastanoi* | 0.065 | 0.077 |
| *Pseudomonas sediminis* | 0.159 | 0.075 |
| *Pseudomonas sessilinigenes* | 0.000 | 0.007 |
| *Pseudomonas shahriarae* | 0.000 | 0.005 |
| *Pseudomonas sihuiensis* | 0.098 | 0.561 |
| *Pseudomonas silesiensis* | 0.149 | 0.382 |
| *Pseudomonas simiae* | 0.490 | 0.132 |
| *Pseudomonas soli* | 0.136 | 0.013 |
| *Pseudomonas* sp. 02C 26 | 0.000 | 0.036 |
| *Pseudomonas* sp. 09C 129 | 0.000 | 0.012 |
| *Pseudomonas* sp. 13159349 | 0.000 | 0.005 |
| *Pseudomonas* sp. 14181154 | 0.000 | 0.127 |
| *Pseudomonas* sp. 15A4 | 0.470 | 0.173 |
| *Pseudomonas* sp. 2hn | 0.026 | 0.056 |
| *Pseudomonas* sp. 31-12 | 0.207 | 0.019 |
| *Pseudomonas* sp. 3-2 | 0.000 | 0.006 |
| *Pseudomonas* sp. 7-41 | 0.000 | 0.026 |
| *Pseudomonas* sp. 7SR1 | 0.140 | 0.061 |
| *Pseudomonas* sp. A2 | 0.000 | 0.048 |
| *Pseudomonas* sp. A214 | 0.000 | 0.013 |
| *Pseudomonas* sp. ABC1 | 0.452 | 0.708 |
| *Pseudomonas* sp. ACM7 | 0.019 | 0.110 |
| *Pseudomonas* sp. ADAK13 | 0.084 | 0.042 |
| *Pseudomonas* sp. ADAK18 | 0.230 | 0.056 |
| *Pseudomonas* sp. ADAK22 | 0.030 | 0.011 |
| *Pseudomonas* sp. ADPe | 0.027 | 0.050 |
| *Pseudomonas* sp. AN-B15 | 0.085 | 0.034 |
| *Pseudomonas* sp. AO-1 | 0.000 | 0.059 |
| *Pseudomonas* sp. ArH3a | 0.030 | 0.000 |
| *Pseudomonas* sp. ATCC 13867 | 0.131 | 0.122 |
| *Pseudomonas* sp. ATCC 43928 | 0.000 | 0.006 |
| *Pseudomonas* sp. B10 | 0.000 | 0.013 |
| *Pseudomonas* sp. B11D7D | 0.249 | 0.221 |
| *Pseudomonas* sp. B14-6 | 0.000 | 0.049 |
| *Pseudomonas* sp. BC42 | 0.000 | 0.017 |
| *Pseudomonas* sp. BIGb0427 | 0.000 | 0.007 |
| *Pseudomonas* sp. BIOMIG1BAC | 0.086 | 0.019 |
| *Pseudomonas* sp. BJP69 | 0.000 | 0.005 |
| *Pseudomonas* sp. C27(2019) | 0.339 | 0.715 |
| *Pseudomonas* sp. Cab53 | 0.027 | 0.014 |
| *Pseudomonas* sp. CC6-YY-74 | 0.751 | 0.486 |
| *Pseudomonas* sp. CCOS 191 | 0.000 | 0.017 |
| *Pseudomonas* sp. CFA | 0.027 | 0.000 |
| *Pseudomonas* sp. CFSAN084952 | 0.054 | 0.000 |
| *Pseudomonas* sp. CIP-10 | 0.038 | 0.000 |
| *Pseudomonas* sp. CMR5c | 0.205 | 0.045 |
| *Pseudomonas* sp. CYM-20-01 | 0.796 | 0.045 |
| *Pseudomonas* sp. DG56-2 | 0.646 | 0.197 |
| *Pseudomonas* sp. DNDY-54 | 0.046 | 0.064 |
| *Pseudomonas* sp. DR 5-09 | 0.027 | 0.000 |
| *Pseudomonas* sp. DR48 | 0.354 | 0.047 |
| *Pseudomonas* sp. DTU12.1 | 0.057 | 0.049 |
| *Pseudomonas* sp. DTU12.3 | 0.072 | 0.012 |
| *Pseudomonas* sp. DY-1 | 0.547 | 0.287 |
| *Pseudomonas* sp. Eqa60 | 0.023 | 0.000 |
| *Pseudomonas* sp. FDAARGOS_380 | 0.348 | 0.006 |
| *Pseudomonas* sp. FGI182 | 0.000 | 0.033 |
| *Pseudomonas* sp. FIT81 | 0.054 | 0.048 |
| *Pseudomonas* sp. gcc21 | 0.598 | 0.727 |
| *Pseudomonas* sp. GR 6-02 | 0.000 | 0.024 |
| *Pseudomonas* sp. HLS-6 | 0.383 | 0.152 |
| *Pseudomonas* sp. HN11 | 0.000 | 0.121 |
| *Pseudomonas* sp. HN2 | 0.286 | 0.012 |
| *Pseudomonas* sp. HN2-3 | 0.023 | 0.065 |
| *Pseudomonas* sp. HN8-3 | 0.000 | 0.035 |
| *Pseudomonas* sp. HS-18 | 0.056 | 0.333 |
| *Pseudomonas* sp. I3-I5 | 0.026 | 0.000 |
| *Pseudomonas* sp. IAC-BECa141 | 0.023 | 0.000 |
| *Pseudomonas* sp. IB20 | 0.049 | 0.000 |
| *Pseudomonas* sp. IzPS59 | 0.087 | 0.016 |
| *Pseudomonas* sp. J380 | 0.000 | 0.178 |
| *Pseudomonas* sp. KNUC1026 | 0.820 | 0.212 |
| *Pseudomonas* sp. KUIN-1 | 0.023 | 0.000 |
| *Pseudomonas* sp. L5B5 | 0.509 | 0.615 |
| *Pseudomonas* sp. LAB-08 | 0.321 | 0.173 |
| *Pseudomonas* sp. LBUM920 | 0.030 | 0.005 |
| *Pseudomonas* sp. Leaf58 | 0.273 | 0.058 |
| *Pseudomonas* sp. LG1E9 | 0.359 | 0.081 |
| *Pseudomonas* sp. LH1G9 | 0.000 | 0.007 |
| *Pseudomonas* sp. LPB0260 | 0.668 | 0.468 |
| *Pseudomonas* sp. LPH1 | 0.158 | 0.095 |
| *Pseudomonas* sp. LS.1a | 0.133 | 0.027 |
| *Pseudomonas* sp. LTJR-52 | 0.082 | 0.162 |
| *Pseudomonas* sp. M1 | 0.000 | 0.189 |
| *Pseudomonas* sp. M30-35 | 0.866 | 0.391 |
| *Pseudomonas* sp. Marseille-Q3773 | 0.276 | 0.172 |
| *Pseudomonas* sp. MM211 | 0.669 | 0.156 |
| *Pseudomonas* sp. MM213 | 0.054 | 0.038 |
| *Pseudomonas* sp. MPC6 | 0.210 | 0.014 |
| *Pseudomonas* sp. MPDS | 0.027 | 0.071 |
| *Pseudomonas* sp. MPFS | 0.060 | 0.010 |
| *Pseudomonas* sp. MRSN12121 | 0.000 | 0.007 |
| *Pseudomonas* sp. MSPm1 | 0.365 | 0.089 |
| *Pseudomonas* sp. MTM4 | 0.162 | 0.280 |
| *Pseudomonas* sp. MYb193 | 0.000 | 0.023 |
| *Pseudomonas* sp. NIBRBAC000502773 | 0.106 | 0.034 |
| *Pseudomonas* sp. NIBR-H-19 | 0.075 | 0.046 |
| *Pseudomonas* sp. NS1(2017) | 0.056 | 0.016 |
| *Pseudomonas* sp. OE 28.3 | 0.026 | 0.161 |
| *Pseudomonas* sp. OIL-1 | 0.399 | 0.839 |
| *Pseudomonas* sp. Os17 | 0.000 | 0.005 |
| *Pseudomonas* sp. OST1909 | 0.000 | 0.007 |
| *Pseudomonas* sp. Ost2 | 0.081 | 0.089 |
| *Pseudomonas* sp. p1(2021b) | 0.441 | 0.172 |
| *Pseudomonas* sp. Pc102 | 0.119 | 0.296 |
| *Pseudomonas* sp. PDNC002 | 0.170 | 0.158 |
| *Pseudomonas* sp. phDV1 | 0.171 | 0.127 |
| *Pseudomonas* sp. PONIH3 | 0.000 | 0.034 |
| *Pseudomonas* sp. PP3 | 0.026 | 0.565 |
| *Pseudomonas* sp. R2-37-08W | 0.026 | 0.048 |
| *Pseudomonas* sp. R2-7-07 | 0.057 | 0.007 |
| *Pseudomonas* sp. R2A2 | 0.302 | 0.065 |
| *Pseudomonas* sp. R3-18-08 | 0.056 | 0.008 |
| *Pseudomonas* sp. R32 | 0.046 | 0.000 |
| *Pseudomonas* sp. R3-52-08 | 0.049 | 0.006 |
| *Pseudomonas* sp. R4-35-07 | 0.000 | 0.026 |
| *Pseudomonas* sp. R4-39-08 | 0.027 | 0.000 |
| *Pseudomonas* sp. R5-89-07 | 0.590 | 0.084 |
| *Pseudomonas* sp. R76 | 0.000 | 0.087 |
| *Pseudomonas* sp. R84 | 0.000 | 0.034 |
| *Pseudomonas* sp. RBPA9 | 0.000 | 0.019 |
| *Pseudomonas* sp. RC3H12 | 0.263 | 0.135 |
| *Pseudomonas* sp. RtIB026 | 0.119 | 0.665 |
| *Pseudomonas* sp. RU47 | 0.054 | 0.000 |
| *Pseudomonas* sp. S07E 245 | 0.026 | 0.013 |
| *Pseudomonas* sp. S09G 359 | 0.080 | 0.024 |
| *Pseudomonas* sp. S150 | 0.131 | 0.059 |
| *Pseudomonas* sp. S34 | 0.130 | 0.049 |
| *Pseudomonas* sp. S35 | 0.329 | 0.014 |
| *Pseudomonas* sp. S49 | 0.108 | 0.000 |
| *Pseudomonas* sp. SC3(2021) | 0.023 | 0.007 |
| *Pseudomonas* sp. SCA2728.1_7 | 0.912 | 0.013 |
| *Pseudomonas* sp. SCB32 | 0.211 | 0.073 |
| *Pseudomonas* sp. SDM007 | 0.032 | 0.024 |
| *Pseudomonas* sp. Seg1 | 0.053 | 0.020 |
| *Pseudomonas* sp. SGAir0191 | 0.230 | 0.041 |
| *Pseudomonas* sp. SK | 0.065 | 0.340 |
| *Pseudomonas* sp. SK2 | 0.023 | 0.000 |
| *Pseudomonas* sp. SK3(2021) | 0.060 | 0.000 |
| *Pseudomonas* sp. So3.2b | 0.019 | 0.005 |
| *Pseudomonas* sp. SORT22 | 0.000 | 0.030 |
| *Pseudomonas* sp. St29 | 0.057 | 0.021 |
| *Pseudomonas* sp. St316 | 0.000 | 0.156 |
| *Pseudomonas* sp. StFLB209 | 0.523 | 0.333 |
| *Pseudomonas* sp. SWI6 | 0.000 | 0.029 |
| *Pseudomonas* sp. SXM-1 | 0.023 | 0.062 |
| *Pseudomonas* sp. TCU-HL1 | 0.477 | 0.390 |
| *Pseudomonas* sp. THAF7b | 0.248 | 0.014 |
| *Pseudomonas* sp. TKP | 0.000 | 0.007 |
| *Pseudomonas* sp. Tri1 | 0.082 | 0.019 |
| *Pseudomonas* sp. URMO17WK12:I11 | 0.388 | 0.048 |
| *Pseudomonas* sp. UW4 | 0.109 | 0.007 |
| *Pseudomonas* sp. VLB120 | 0.027 | 0.000 |
| *Pseudomonas* sp. WCS374 | 0.023 | 0.000 |
| *Pseudomonas* sp. Y39-6 | 0.030 | 0.000 |
| *Pseudomonas* sp. Y5-11 | 0.000 | 0.846 |
| *Pseudomonas* sp. Z003-0.4C(8344-21) | 0.027 | 0.007 |
| *Pseudomonas stutzeri* | 8.376 | 3.592 |
| *Pseudomonas synxantha* | 1.213 | 0.087 |
| *Pseudomonas syringae* | 0.782 | 0.241 |
| *Pseudomonas syringae* group genomosp. 7 | 0.436 | 0.114 |
| *Pseudomonas taetrolens* | 1.855 | 0.195 |
| *Pseudomonas tensinigenes* | 0.080 | 0.022 |
| *Pseudomonas thivervalensis* | 0.070 | 0.034 |
| *Pseudomonas tohonis* | 0.120 | 0.068 |
| *Pseudomonas tolaasii* | 0.140 | 0.122 |
| *Pseudomonas toyotomiensis* | 0.000 | 0.336 |
| *Pseudomonas tritici* | 0.237 | 0.029 |
| *Pseudomonas trivialis* | 0.200 | 0.156 |
| *Pseudomonas tructae* | 0.087 | 0.054 |
| *Pseudomonas umsongensis* | 0.102 | 0.108 |
| *Pseudomonas vancouverensis* | 1.596 | 0.261 |
| *Pseudomonas vanderleydeniana* | 0.167 | 0.084 |
| *Pseudomonas veronii* | 0.164 | 0.160 |
| *Pseudomonas versuta* | 0.351 | 0.064 |
| *Pseudomonas viciae* | 1.014 | 0.059 |
| *Pseudomonas viridiflava* | 0.711 | 0.312 |
| *Pseudomonas wenzhouensis* | 0.313 | 0.112 |
| *Pseudomonas xantholysinigenes* | 0.180 | 0.039 |
| *Pseudomonas xanthomarina* | 0.335 | 0.294 |
| *Pseudomonas xanthosomae* | 0.098 | 0.046 |
| *Pseudomonas yamanorum* | 0.668 | 0.145 |
| *Pseudomonas zarinae* | 0.034 | 0.005 |
| *Pseudomonas zeae* | 0.303 | 0.041 |
| *Pseudomonas zhaodongensis* | 0.312 | 0.411 |

Table S-22: *Rickettsia* species detected.

| **Species Name** | **STS Average (ppm)** | **LTS Average (ppm)** |
| --- | --- | --- |
| *Rickettsia* endosymbiont of Ixodes scapularis | 0.049 | 0.000 |
| *Rickettsia prowazekii* | 0.059 | 0.006 |
| *Rickettsia rhipicephali* | 0.027 | 0.000 |
| *Rickettsia tillamookensis* | 0.054 | 0.000 |

Table S-23: Salmonella species detected.

| **Species Name** | **STS Average (ppm)** | **LTS Average (ppm)** |
| --- | --- | --- |
| *Salmonella bongori* | 1.356 | 0.020 |
| *Salmonella enterica* | 5.692 | 0.133 |

Table S-24: *Shigella* species detected.

| **Species Name** | **STS Average (ppm)** | **LTS Average (ppm)** |
| --- | --- | --- |
| *Shigella boydii* | 0.049 | 0.006 |
| *Shigella dysenteriae* | 0.046 | 0.000 |
| *Shigella flexneri* | 0.487 | 0.000 |
| *Shigella sonnei* | 0.019 | 0.000 |

Table S-25: *Staphylococcus* species detected.

| **Species Name** | **STS Average (ppm)** | **LTS Average (ppm)** |
| --- | --- | --- |
| *Staphylococcus arlettae* | 0.056 | 0.031 |
| *Staphylococcus aureus* | 0.138 | 5.537 |
| *Staphylococcus auricularis* | 0.059 | 0.006 |
| *Staphylococcus capitis* | 1.637 | 1.028 |
| *Staphylococcus caprae* | 0.339 | 16.499 |
| *Staphylococcus carnosus* | 0.056 | 0.226 |
| *Staphylococcus chromogenes* | 0.026 | 0.032 |
| *Staphylococcus cohnii* | 0.651 | 0.092 |
| *Staphylococcus condimenti* | 0.053 | 0.221 |
| *Staphylococcus debuckii* | 0.027 | 0.038 |
| *Staphylococcus delphini* | 11.535 | 0.045 |
| *Staphylococcus epidermidis* | 4.801 | 7.642 |
| *Staphylococcus equorum* | 0.085 | 0.121 |
| *Staphylococcus felis* | 0.234 | 0.017 |
| *Staphylococcus gallinarum* | 0.483 | 0.878 |
| *Staphylococcus haemolyticus* | 1.688 | 5.784 |
| *Staphylococcus hominis* | 9.743 | 24.299 |
| *Staphylococcus kloosii* | 0.000 | 3.939 |
| *Staphylococcus lugdunensis* | 0.136 | 0.292 |
| *Staphylococcus lutrae* | 0.030 | 0.000 |
| *Staphylococcus muscae* | 0.000 | 0.014 |
| *Staphylococcus nepalensis* | 0.190 | 0.024 |
| *Staphylococcus pasteuri* | 0.190 | 0.080 |
| *Staphylococcus pettenkoferi* | 0.589 | 0.079 |
| *Staphylococcus piscifermentans* | 0.000 | 0.013 |
| *Staphylococcus pseudintermedius* | 0.000 | 0.020 |
| *Staphylococcus pseudoxylosus* | 0.038 | 0.054 |
| *Staphylococcus roterodami* | 0.053 | 1.066 |
| *Staphylococcus saccharolyticus* | 0.027 | 0.068 |
| *Staphylococcus saprophyticus* | 0.000 | 0.610 |
| *Staphylococcus schleiferi* | 0.328 | 0.033 |
| *Staphylococcus simiae* | 0.161 | 1.850 |
| *Staphylococcus simulans* | 0.000 | 0.012 |
| *Staphylococcus* sp. 11-B-312 | 0.094 | 0.045 |
| *Staphylococcus* sp. 17KM0847 | 0.150 | 1.419 |
| *Staphylococcus* sp. CCM 9025 | 0.057 | 0.006 |
| *Staphylococcus* sp. M0911 | 0.000 | 0.014 |
| *Staphylococcus* sp. MI 10-1553 | 0.000 | 0.022 |
| *Staphylococcus* sp. SB1-57 | 0.000 | 0.038 |
| *Staphylococcus* sp. T93 | 0.026 | 0.000 |
| *Staphylococcus succinus* | 0.034 | 0.018 |
| *Staphylococcus warneri* | 0.000 | 0.007 |
| *Staphylococcus xylosus* | 0.000 | 0.047 |

Table S-26: *Vibrio* species detected.

| **Species Name** | **STS Average (ppm)** | **LTS Average (ppm)** |
| --- | --- | --- |
| *Vibrio alfacsensis* | 157.626 | 4.362 |
| *Vibrio algicola* | 40.589 | 0.177 |
| *Vibrio alginolyticus* | 110250.627 | 2041.401 |
| *Vibrio anguillarum* | 1697.132 | 12.359 |
| *Vibrio antiquarius* | 51.486 | 0.721 |
| *Vibrio aphrogenes* | 78.479 | 0.244 |
| *Vibrio aquimaris* | 357.250 | 9.121 |
| *Vibrio astriarenae* | 166.270 | 1.927 |
| *Vibrio atlanticus* | 115.190 | 1.533 |
| *Vibrio azureus* | 406.731 | 2.924 |
| *Vibrio bathopelagicus* | 95.368 | 1.430 |
| *Vibrio breoganii* | 69.540 | 0.363 |
| *Vibrio campbellii* | 2456.147 | 147.231 |
| *Vibrio casei* | 962.719 | 3.613 |
| *Vibrio chagasii* | 387.059 | 30.599 |
| *Vibrio cholerae* | 9292.942 | 190.094 |
| *Vibrio cidicii* | 117.986 | 1.324 |
| *Vibrio cincinnatiensis* | 643.135 | 2.617 |
| *Vibrio coralliilyticus* | 21692.858 | 856.654 |
| *Vibrio crassostreae* | 180.508 | 3.711 |
| *Vibrio cyclitrophicus* | 338.075 | 3.347 |
| *Vibrio diabolicus* | 6530.336 | 72.307 |
| *Vibrio europaeus* | 163.009 | 76.832 |
| *Vibrio fluvialis* | 4790.520 | 23.409 |
| *Vibrio furnissii* | 697.878 | 5.998 |
| *Vibrio gazogenes* | 42.333 | 0.149 |
| *Vibrio gigantis* | 32.330 | 0.903 |
| *Vibrio harveyi* | 10572.868 | 828.350 |
| *Vibrio hyugaensis* | 144.418 | 2.114 |
| *Vibrio kanaloae* | 205.254 | 1.936 |
| *Vibrio maritimus* | 447.690 | 86.355 |
| *Vibrio mediterranei* | 481.889 | 35.345 |
| *Vibrio metoecus* | 61.321 | 0.212 |
| *Vibrio metschnikovii* | 445.331 | 1.198 |
| *Vibrio mimicus* | 2117.190 | 3.969 |
| *Vibrio natriegens* | 851.959 | 5.640 |
| *Vibrio navarrensis* | 371.069 | 26.122 |
| *Vibrio neocaledonicus* | 411.579 | 9.873 |
| *Vibrio neptunius* | 255.666 | 150.421 |
| *Vibrio nigripulchritudo* | 409.238 | 7.375 |
| *Vibrio owensii* | 10412.985 | 185.556 |
| *Vibrio panuliri* | 183.030 | 0.920 |
| *Vibrio paracholerae* | 26.477 | 0.369 |
| *Vibrio parahaemolyticus* | 15181.508 | 183.201 |
| *Vibrio penaeicida* | 1616.751 | 11.747 |
| *Vibrio ponticus* | 98.787 | 2.836 |
| *Vibrio qinghaiensis* | 69.476 | 0.388 |
| *Vibrio rotiferianus* | 2476.497 | 19.630 |
| *Vibrio rumoiensis* | 312.697 | 1.261 |
| *Vibrio scophthalmi* | 116.266 | 1.254 |
| *Vibrio sinaloensis* | 196.539 | 29.317 |
| *Vibrio* sp. | 6.594 | 0.597 |
| *Vibrio* sp. 04Ya090 | 0.034 | 0.000 |
| *Vibrio* sp. 09022 | 2.042 | 0.049 |
| *Vibrio* sp. 0908 | 0.941 | 0.113 |
| *Vibrio* sp. 2521-89 | 15.363 | 0.106 |
| *Vibrio* sp. 41 | 0.159 | 0.000 |
| *Vibrio* sp. B1ASS3 | 1163.752 | 18.655 |
| *Vibrio* sp. B1FIG11 | 701.954 | 9.343 |
| *Vibrio* sp. B1FLJ16 | 341.337 | 1.913 |
| *Vibrio* sp. B1REV9 | 39.791 | 0.376 |
| *Vibrio* sp. dhg | 1704.749 | 38.979 |
| *Vibrio* sp. ED004 | 291.690 | 2.411 |
| *Vibrio* sp. EJY3 | 266.675 | 2.344 |
| *Vibrio* sp. HDW18 | 86.131 | 0.200 |
| *Vibrio* sp. OG9-811 | 251.277 | 4.133 |
| *Vibrio* sp. Scap24 | 28.026 | 0.386 |
| *Vibrio* sp. SCSIO 43132 | 12.927 | 0.907 |
| *Vibrio* sp. sp1 | 1195.821 | 10.815 |
| *Vibrio* sp. SS-MA-C1-2 | 20.481 | 0.064 |
| *Vibrio* sp. THAF190c | 26838.209 | 561.879 |
| *Vibrio* sp. THAF191c | 0.030 | 0.000 |
| *Vibrio* sp. VB16 | 48.193 | 0.147 |
| *Vibrio spartinae* | 486.822 | 1.856 |
| *Vibrio splendidus* | 128.545 | 1.741 |
| *Vibrio taketomensis* | 53.265 | 0.208 |
| *Vibrio tritonius* | 130.813 | 0.602 |
| *Vibrio vulnificus* | 2878.408 | 26.313 |
| *Vibrio zhugei* | 1124.714 | 8.066 |
| *Vibrio ziniensis* | 90.631 | 0.339 |

Table S-27: Top species overall in STS and LTS samples

| **Species Name** | **STS Average (ppm)** | **LTS Average (ppm)** |
| --- | --- | --- |
| *Alteromonas macleodii* | 2688.889 | 0.000 |
| *Vibrio alginolyticus* | 1100.000 | 0.000 |
| *Tritonibacter mobilis* | 800.000 | 0.000 |
| *Salipiger sp. CCB-MM3* | 511.111 | 0.000 |
| *Mesoflavibacter sp. SCSIO 43206* | 566.667 | 0.000 |
| *Tenacibaculum mesophilum* | 500.000 | 0.000 |
| *Vibrio sp. THAF190c* | 255.556 | 0.000 |
| *Vibrio coralliilyticus* | 200.000 | 0.000 |
| *Cobetia sp. cqz5-12* | 155.556 | 0.000 |
| *Pseudoalteromonas shioyasakiensis* | 200.000 | 0.000 |
| *Alteromonas sp. BL110* | 244.444 | 0.000 |
| *Pseudoalteromonas spongiae* | 200.000 | 0.000 |
| *Vibrio parahaemolyticus* | 155.556 | 0.000 |
| *Alteromonas mediterranea* | 144.444 | 0.000 |
| *Halomonas sp. THAF12* | 100.000 | 0.000 |
| *Seonamhaeicola sp. S2-3* | 100.000 | 0.000 |
| *Pseudoalteromonas piscicida* | 100.000 | 0.000 |
| *Vibrio harveyi* | 100.000 | 0.000 |
| *Vibrio owensii* | 100.000 | 0.000 |
| *Vibrio cholerae* | 100.000 | 0.000 |
| *Microbulbifer thermotolerans* | 0.000 | 6236.364 |
| *Oricola thermophila* | 0.000 | 2927.273 |
| *Thermoactinomyces vulgaris* | 0.000 | 100.000 |
| *Bacillus paralicheniformis* | 0.000 | 100.000 |
